# Reduced thyroxine production in young household contacts of tuberculosis patients increases active tuberculosis disease risk

**DOI:** 10.1172/jci.insight.148271

**Published:** 2021-07-08

**Authors:** Kamakshi Prudhula Devalraju, Deepak Tripathi, Venkata Sanjeev Kumar Neela, Padmaja Paidipally, Rajesh Kumar Radhakrishnan, Karan P. Singh, Mohammad Soheb Ansari, Martin Jaeger, Romana T. Netea-Maier, Mihai G. Netea, Sunmi Park, Sheue-yann Cheng, Vijaya Lakshmi Valluri, Ramakrishna Vankayalapati

**Affiliations:** 1 Immunology and Molecular Biology Department, Bhagwan Mahavir Medical Research Centre, Hyderabad, Telangana, India.; 2 Department of Pulmonary Immunology, Center for Biomedical Research, University of Texas Health Science Center, Tyler, Texas, USA.; 3 Department of Epidemiology and Biostatistics, School of Community and Rural Health, University of Texas Health Science Center, Tyler, Texas, USA.; 4 Department of Internal Medicine, Division of Endocrinology, and; 5 Department of Internal Medicine and Radboud Center for Infectious Diseases, Radboud University Medical Center, Nijmegen, Netherlands.; 6 Laboratory of Molecular Biology, National Cancer Institute, NIH, Bethesda, Maryland, USA.

**Keywords:** Immunology, Pulmonology, Bacterial infections, Cytokines

## Abstract

In the current study, we followed 839 household contacts (HHCs) of tuberculosis (TB) patients for 2 years and identified the factors that enhanced the development of TB. Fourteen of the 17 HHCs who progressed to TB were in the 15- to 30-year-old age group. At baseline (the “0“ time point, when all the individuals were healthy), the concentration of the thyroid hormone thyroxine (T4) was lower, and there were increased numbers of Tregs in PBMCs of TB progressors. At baseline, PBMCs from TB progressors stimulated with early secretory antigenic target 6 (ESAT-6) and 10 kDa culture filtrate antigen (CFP-10) produced less IL-1α. Thyroid hormones inhibited *Mycobacterium tuberculosis* (Mtb) growth in macrophages in an IL-1α–dependent manner. Mtb-infected Thra1^PV/+^ (mutant thyroid hormone receptor) mice had increased mortality and reduced IL-1α production. Our findings suggest that young HHCs who exhibit decreased production of thyroid hormones are at high risk of developing active TB disease.

## Graphical abstract

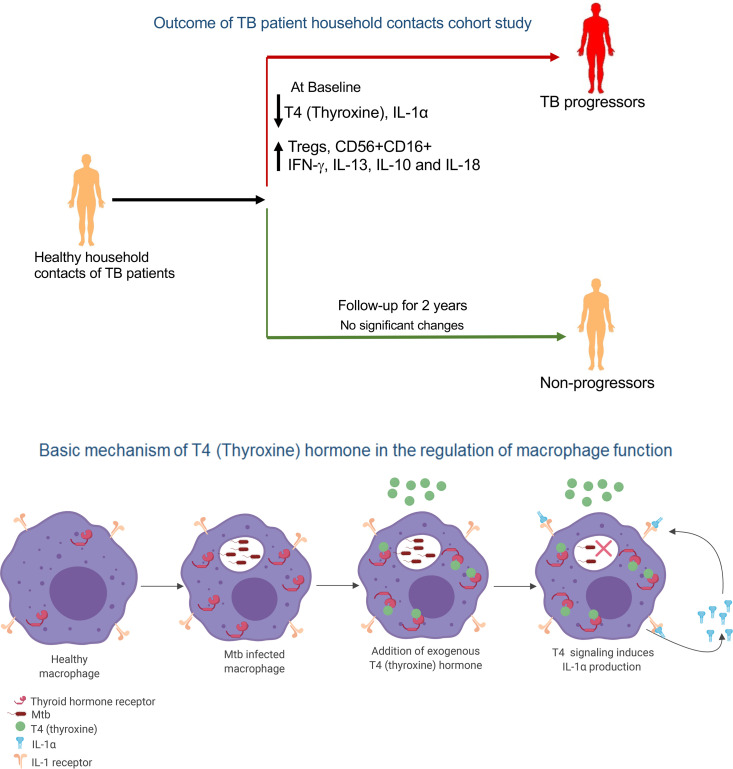

## Introduction

*Mycobacterium tuberculosis* (Mtb) infects one-third of humans and causes almost 1.3 million deaths per year (1). Approximately 90% of infected persons have latent tuberculosis infection (LTBI) and remain well, but 10% develop primary tuberculosis (TB) soon after infection or reactivation of TB many years later (2, 3). Nevertheless, only a small percentage of persons with LTBI develop TB and therefore are required to complete treatment regimens; approximately 13%–42% of individuals prescribed treatment, fail to complete therapy (4, 5). The identification of persons with LTBI who are at greatly increased risk of development of TB would be a significant breakthrough that would allow public health resources to be focused on high-risk individuals, facilitate the completion of therapy for LTBI, and prevent the future development of TB. Severe immunosuppression due to HIV infection and treatment with corticosteroids and anti-TNF blockers markedly increase the risk of the progression of LTBI to active TB (6–9). Among persons who are not clinically immunocompromised, limited information is available about other immune mechanisms that favor the progression of LTBI to TB (10–21).

The household contacts (HHCs) of TB patients are at high risk of developing latent Mtb infection or active TB disease (22, 23). Identifying the HHCs that are likely to develop active TB is important to control TB (24–26). Examination of HHCs is a natural method to study active TB disease development in a real-time manner and to explore the early immune components involved in protection against TB. However, a detailed follow-up study and mechanistic understanding are important to identify the healthy HHCs of TB patients who are at high risk.

Immune responses mediated by antigen-presenting cells, NK cells, and T cells play an important role in the control of Mtb infection (27). This is partly achieved through the production of cytokines such as IL-1α, TNF-α, IFN-γ, and IL-1β in the lungs of Mtb-infected mice and independently required for host resistance (28). TNF-α–mediated signaling is critical for reactive nitrogen production in macrophages and protection against Mtb (29). IFN-γ induces macrophage activation and controls growth of intracellular pathogens.

Previous studies have demonstrated that monocytes, NK cells, innate lymphoid cells, and Tregs have the potential to contribute to innate and adaptive immune responses during Mtb infection (30–33). Endocrine abnormalities such as adrenal insufficiency, diabetes mellitus, and calcium-vitamin D abnormalities are common in TB patients, suggesting that the endocrine system also plays an important role in the control of Mtb infection (34–36). Hormones can affect immune cell function and induce the production of inflammatory cytokines. In a rabbit Mtb infection model, it was shown that defective thyroid hormone production enhances susceptibility to Mtb infection (37). However, it is not known whether defective hormone production leads to the development of active TB disease in HHCs of TB patients.

In the current study, we evaluated the phenotype and function of various immune cells and other factors, such as hormones, in a large group of household contacts of TB patients (a total of 839 individuals). We repeated these evaluations at 24 months while monitoring the subjects for the development of active TB. By following a large cohort of HHCs of TB patients, we determined whether the development of TB is preceded by specific changes in the cellular markers of immune cells and other factors. We also confirmed our findings using a mouse model of Mtb infection.

## Results

### Demographic characteristics of the cohort.

Among the 839 individuals enrolled, we had 83.5% retention, and 138 individuals were lost to follow-up. The median number of HHCs per index case was 3. Fifty-nine percent of the HHCs were LTBI-positive at baseline, and 2.53% of the HHCs progressed to active TB disease during follow-up. Among the 839 HHCs, 465 (56%) were 15–30 years old, 228 (27%) were 31–44 years old, and 146 (17%) were 45–73 years old (Table 1). Demographic and clinical characteristics of the study participants are shown in Table 1. The summaries of screening, enrollment, and loss to follow-up are shown in Supplemental Tables 1 and 2; supplemental material available online with this article; https://doi.org/10.1172/jci.insight.148271DS1

### The prevalence of TB was high in young HHCs.

**Figure 1:**
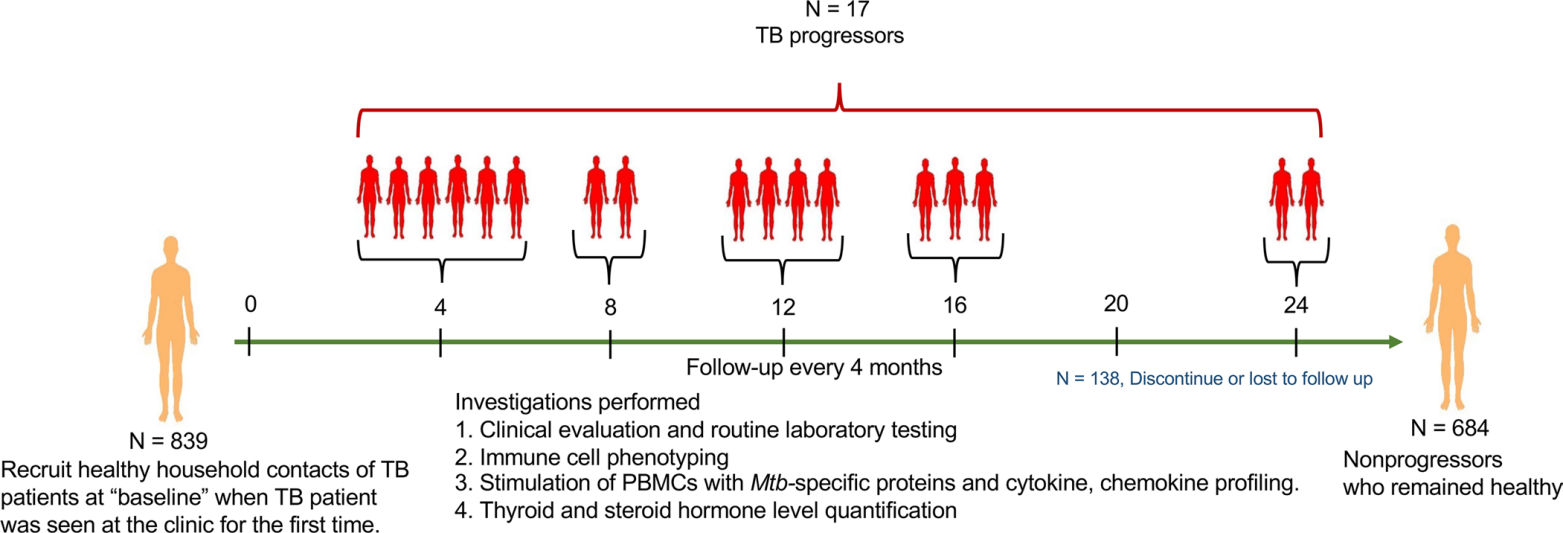
Study design and outcomes for the household contacts in the TB patient cohort study. Schematic representation of the experimental design.

During the 2-year follow-up period, 688 HHCs successfully completed the study. Seventeen (2.53%) out of 688 HHCs developed active TB (progressors), and 671 of the HHCs remained healthy (nonprogressors) (Figure 1 and Table 1). Among the 17 progressors, 14 (82.35%) were in the 15–30-year-old age group, 2 (11.8%) were in the 31–44-year-old age group, and 1 (5.9%) was in the 45–73-year-old age group (Supplemental Table 3). In summary, young HHCs of TB index cases were at increased risk of developing active TB (relative risk, 3.75; 95% CI, 1.086 to 12.96; *P* = 0.036). The demographic and clinical characteristics of the HHCs that developed active TB during the 2-year study are shown in Supplemental Table 3. The complete blood cell counts of the progressors and nonprogressors are shown in Supplemental Figure 1.

### High CD16^+^CD56^+^ and Treg cell frequencies in progressors at baseline.

**Figure 2:**
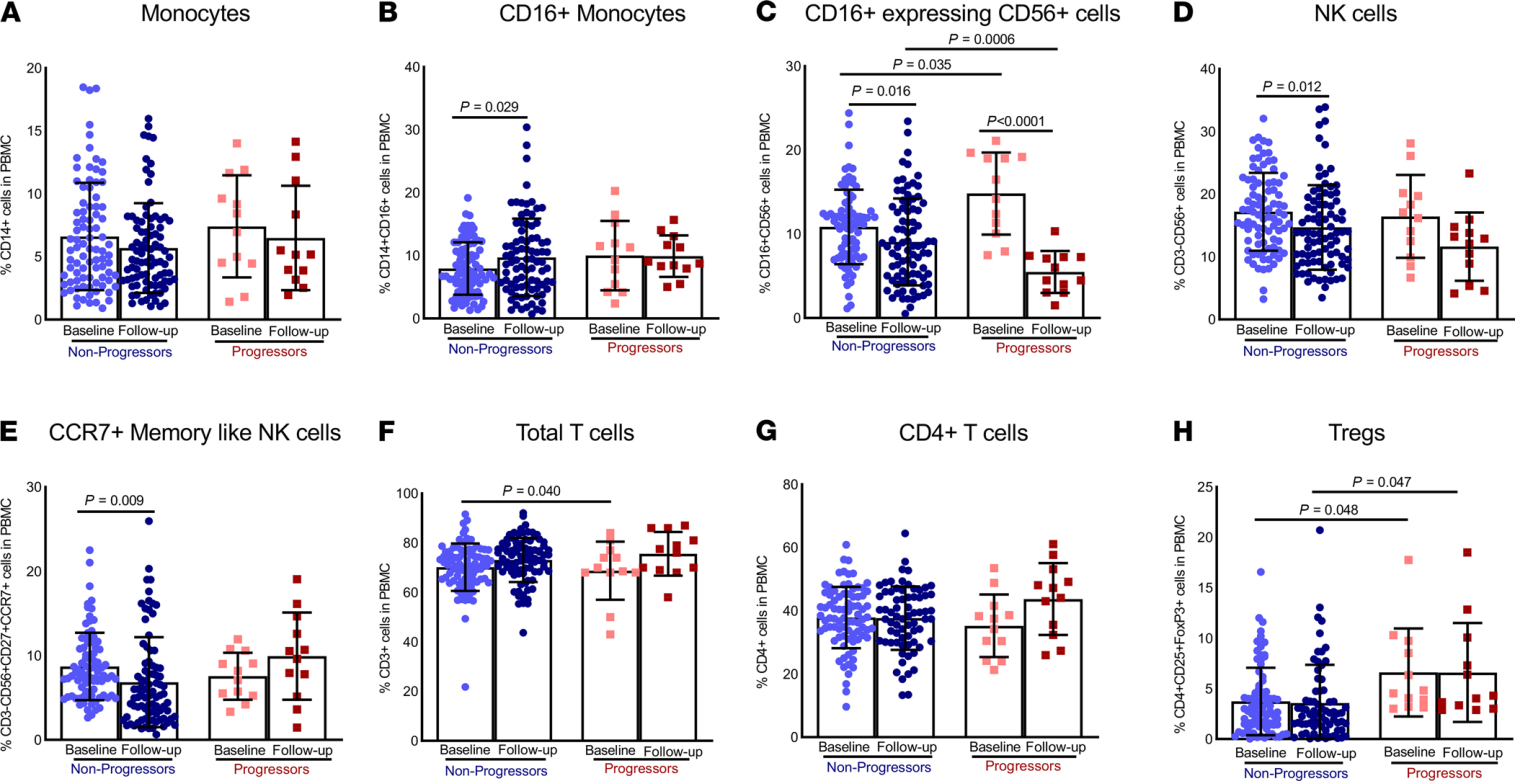
Immune cell distribution in the PBMCs of household contacts of TB patients. PBMCs were isolated from age-matched non-TB progressors (*n* = 87) and TB progressors (*n* = 12) at baseline and follow-up (when the progressors were registered as having active TB), and the percentages of (**A**) CD14^+^, (**B**) CD14^+^CD16^+^, (**C**) CD16^+^CD56^+^, (**D**) CD3^–^CD56^+^, (**E**) CD3^–^CD56^+^CD27^+^CCR7^+^, (**F**) CD3^+^, (**G**) CD4^+^, and (**H**) CD4^+^CD25^+^FoxP3^+^ cells were determined by flow cytometry. The data from 87 nonprogressors and 12 progressors are shown. All the age-matched, non-TB progressors were healthy, nonsmoking, and nonalcoholic and were without any immunosuppressive conditions at baseline or follow-up. Data from smoking and alcoholic progressors were not included. *P* values were determined using 1-way ANOVA with Tukey’s multiple-comparison test. The mean ± SEM and *P* values are shown.

We determined the percentages of various immune cell populations in freshly isolated PBMCs of subsets of nonprogressors (*n* = 87) and progressors (*n* = 12) at baseline and during the follow-up visits. The frequencies of CD14^+^ (*P* = NS, Figure 2A and Supplemental Figure 2) and CD14^+^CD16^+^ cells (*P* = NS, Figure 2B and Supplemental Figure 2) were similar at baseline in the progressors compared with the nonprogressors. The percentages of CD16^+^CD56^+^ cells were higher at baseline in the progressors than in the nonprogressors (*P* = 0.035; Figure 2C and Supplemental Figure 2). Interestingly, there was a 2.5-fold decrease in the percentage of CD16^+^CD56^+^ cells (*P* = 0.0006; Figure 2C) in the progressors at follow-up (blood was obtained when they developed active TB) compared with the baseline values. The percentages of CD4^+^CD25^+^FoxP3^+^ cells (Tregs) were significantly higher in the progressors at baseline (*P* = 0.048) and follow-up (*P* = 0.047; Figure 2H and Supplemental Figure 2) than in nonprogressors. No significant differences were observed in percentages of NK cells and T cells in progressors compared to nonprogressors (Figure 2, D–G).

**Figure 3:**
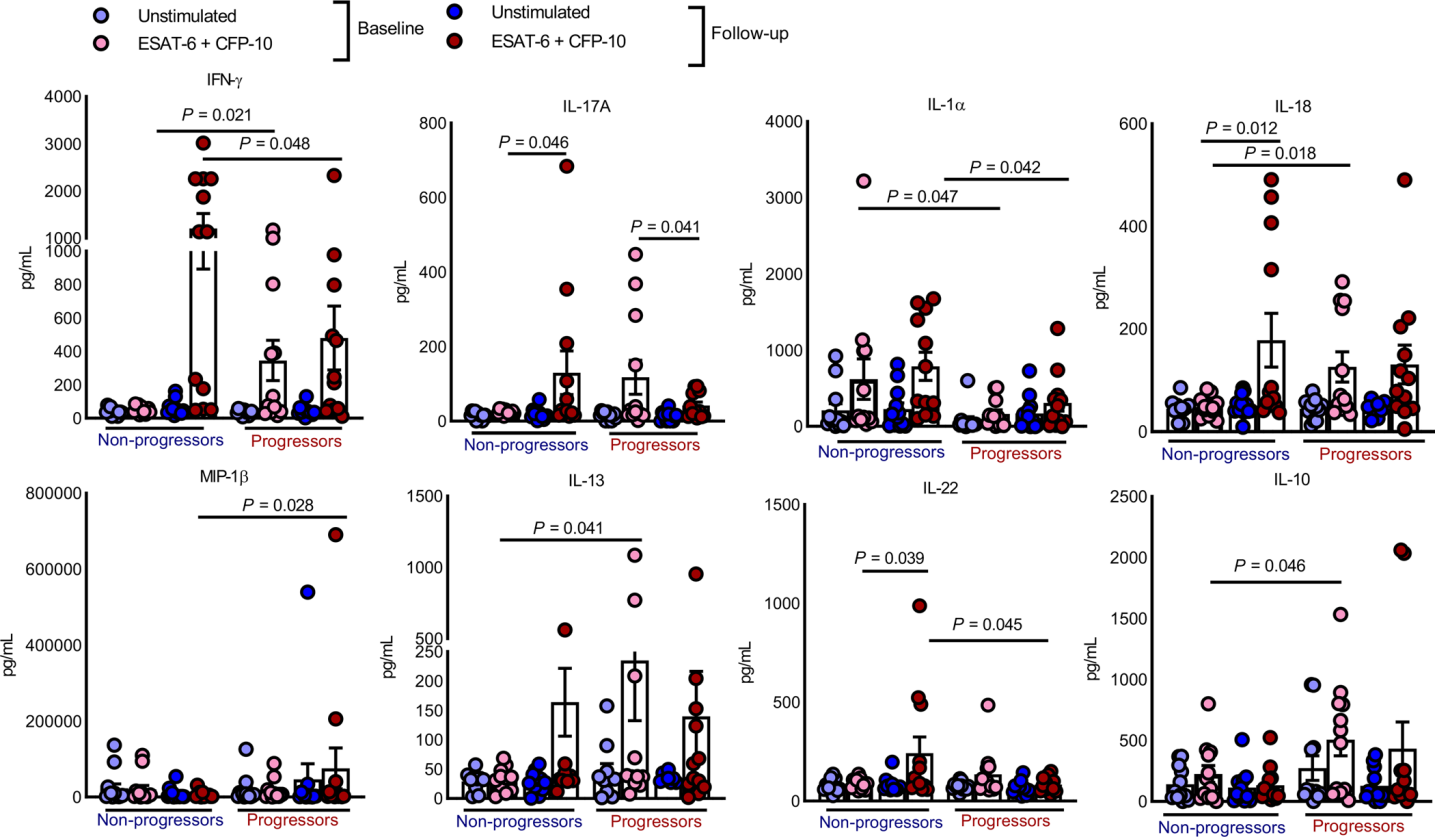
Cytokine and chemokine profiles of ESAT-6– and CFP-10–stimulated PBMCs. PBMCs from nonprogressors (*n* = 12) and progressors (*n* = 12) at baseline and follow-up were isolated and cultured with or without ESAT-6 and CFP-10 (10 μg/mL each), as described in Methods. After 96 hours, the culture supernatants were collected, and levels of various chemokines and cytokines were measured by multiplex ELISA. All the age-matched non-TB progressors were healthy, nonsmoking, and nonalcoholic and were without any immunosuppressive conditions at baseline and follow-up. Data from smoking and alcoholic progressors were not included. *P* values were determined using 1-way ANOVA with Tukey’s multiple-comparison test. The mean values, SEM, and *P* values are shown.

*Reduced IL-1αproduction by PBMCs in progressors at baseline*. We determined cytokine and chemokine production by freshly isolated PBMCs from progressors (*n* = 12) and nonprogressors (*n* = 12) cultured in the presence and absence of the Mtb antigens early secretory antigenic target 6 (ESAT-6) and culture filtrate antigen (CFP-10), as mentioned in the Methods. Among 34 cytokines and chemokines tested, as shown in Figure 3 and Supplemental Figure 3, at baseline, the production of IFN-γ, IL-13, IL-10, and IL-18 was higher in the culture supernatants of the progressors than the nonprogressors (*P* < 0.05, Figure 3). In contrast, IL-1α (*P* = 0.047; Figure 3) levels were significantly lower in the culture supernatants of the progressors compared with the supernatants of the nonprogressors. At follow-up, IFN-γ (*P* = 0.09), IL-17, IL-22, IL-23, and IL-1α (*P* < 0.05) levels were lower in the progressors than in nonprogressors (Figure 3 and Supplemental Figure 3).

### Lower hormone levels in the progressors at baseline.

**Figure 4:**
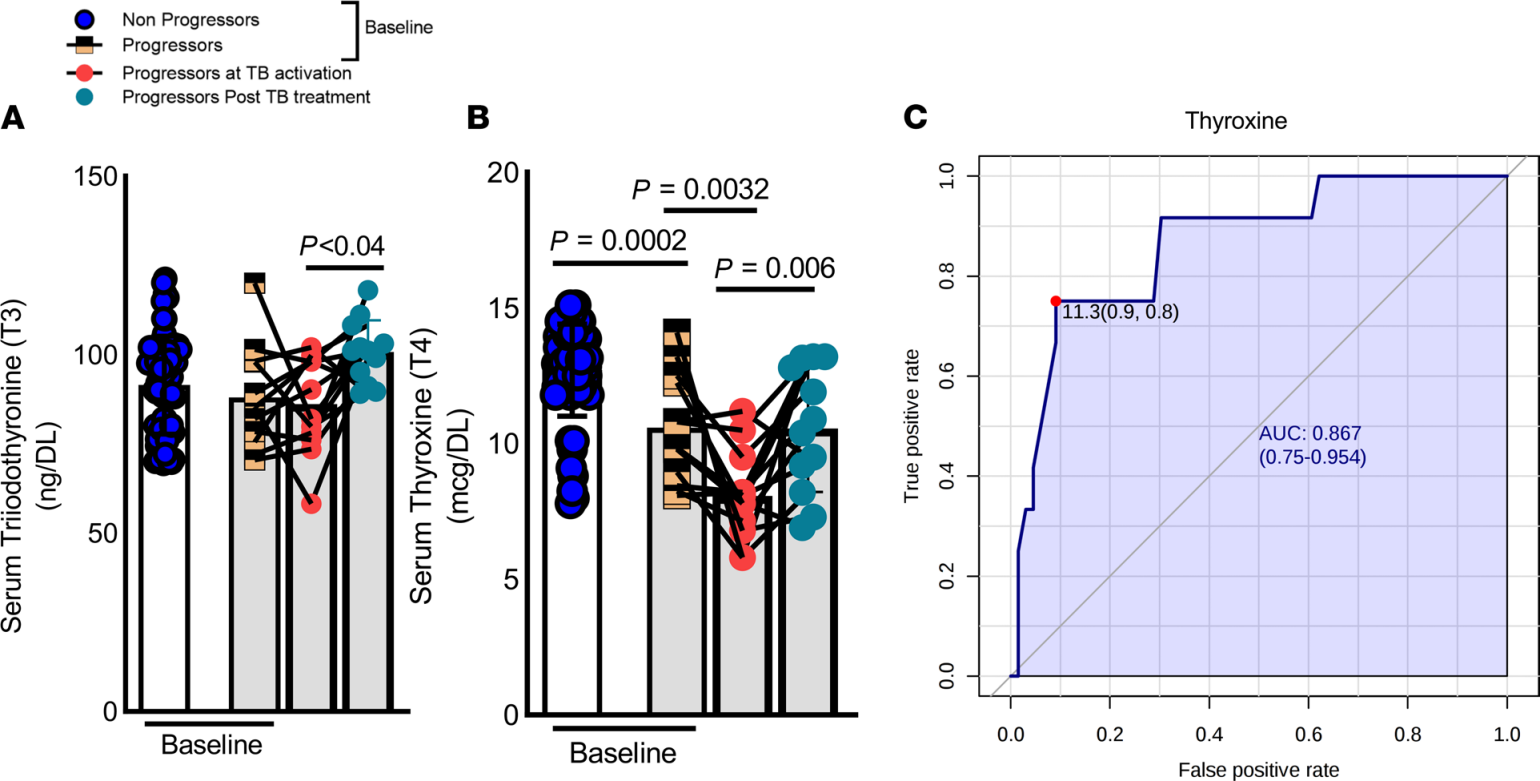
Hormone levels in the serum of HHCs of TB patients. Serum (**A**) T3 and (**B**) T4 hormone levels of the nonprogressors (*n* = 67) and progressors (*n* = 12) at baseline and follow-up (at TB activation) were quantified by ELISA. All the age-matched nonprogressors were healthy, nonsmoking, and nonalcoholic and were without any immunosuppressive conditions at baseline or at follow-up. Data from smoking and alcoholic progressors were not included. *P* values were derived using an unpaired, 2-tailed, independent *t* test. Mean ± SD and *P* values are shown. (**C**) The receiver operating characteristic (ROC) curve for the hormone T4; plot shows the ROC curve for T4 hormone levels in nonprogressors (*n* = 67) and TB progressors (*n* = 12) at baseline. The ROC curve shows the true-positive rate (sensitivity) and false-positive rate (1-specificity) of the model prediction. The biomarker analysis program in MetaboAnalyst software was used to generate the ROC curves and calculate the AUC.

The findings in Supplemental Table 3 indicated that 14 out of the 17 HHCs who developed active TB disease were in the 15- to 30-year-old age group. Given that hormones play an important role in growth, metabolism, and immunity (38), we determined the levels of various serum hormones in the progressors and nonprogressors (both in the same age group) at baseline (Figure 4 and Supplemental Figure 4). Serum dehydroepiandrosterone (DHEA), cortisol, and thyroid stimulating hormone (TSH) levels were similar in progressors and nonprogressors at baseline (Supplemental Figure 4). As shown in Figure 4B, at baseline, the level of thyroxine (T4) hormone (*P* = 0.0002) was significantly lower in the serum of the progressors compared with the nonprogressors (relative risk, 7.3; 95% CI, 3.09 to 17.35; *P* = 0.0001). There was a further decrease in the T4 levels during TB activation (*P* = 0.003) compared with baseline. After standard antitubercular treatment (ATT), serum triiodothyronine (T3) and T4 significantly increased in progressors (Figure 4, A and B). However, 3 years after ATT, the level of T4 hormones significantly decreased in treated progressors (Figure 4B). The diagnostic accuracies of various hormones were evaluated by receiver operating characteristic (ROC) curve analysis to distinguish progressors and nonprogressors at baseline. ROC curve analysis revealed that among all the measured hormones, baseline T4 (AUC > 0.86) and DHEA levels (AUC > 0.78) could distinguish between nonprogressor and progressor HHCs, with a high AUC value (Figure 4C and Supplemental Figure 5, A–C).

**Figure 5:**
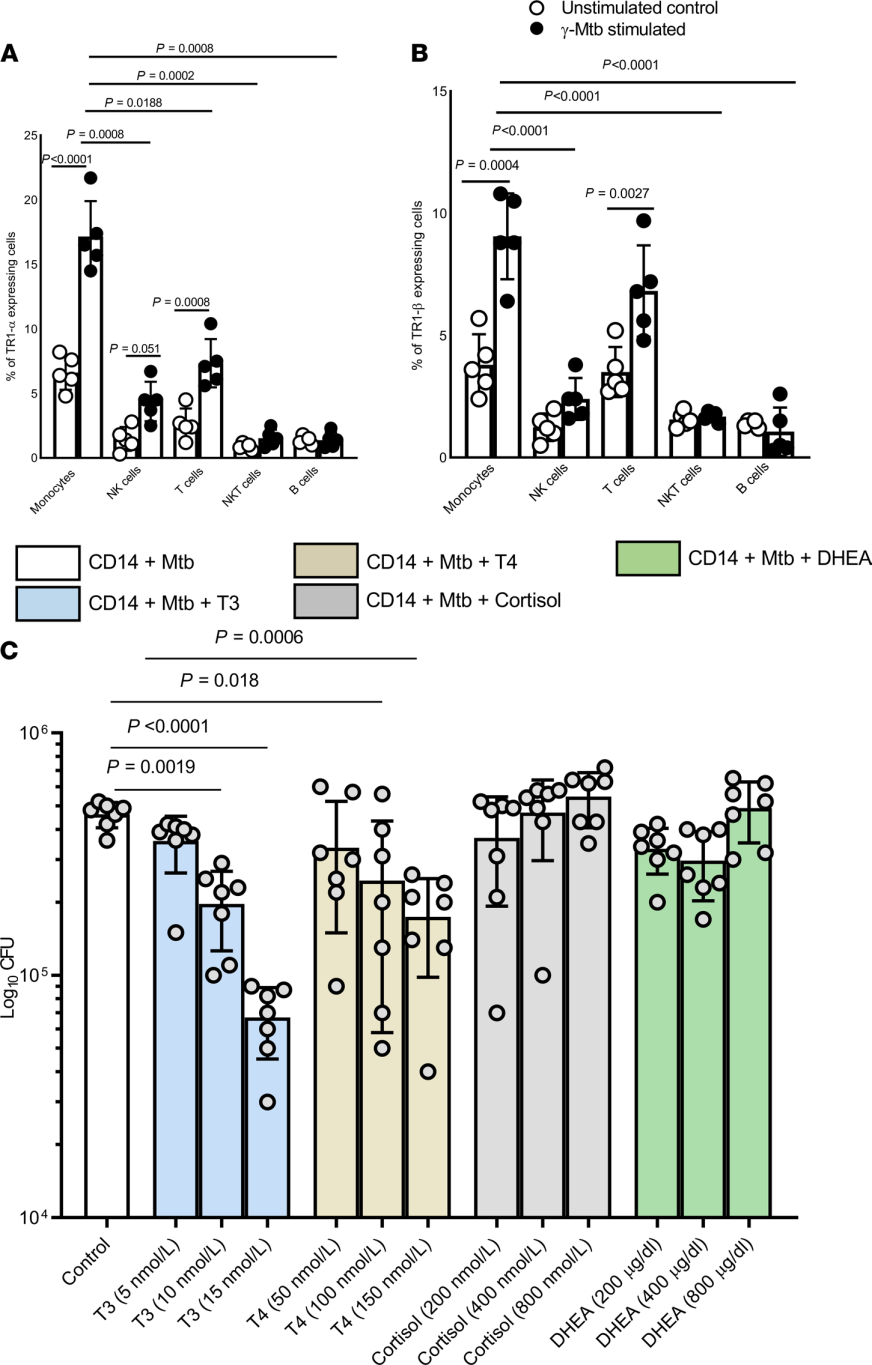
Thyroid hormones restrict Mtb growth in vitro. Freshly isolated PBMCs from 5 healthy individuals (age range: 18–30 years) were cultured with or without γ-irradiated Mtb H37Rv (γ-Mtb, 10 μg/mL) for 120 hours. (**A**) The percentages of TR1-α^+^ and (**B**) TR1-β^+^ cells among monocytes, NK cells, T cells, NKT cells, and B cells were determined by flow cytometry. *P* values were determined using 1-way ANOVA with Tukey’s multiple-comparison test. The mean values, SD, and *P* values are shown. (**C**) Mtb growth in monocyte-derived macrophages (MDMs). The MDMs from healthy individuals (*n* = 7) were infected with H37Rv at an MOI of 2.5 as described in Methods. Some of the infected MDMs were cultured in the presence of T3 (5, 10, 15 nmol/L), T4 (50, 100, 150 nmol/L), DHEA (200, 400, 800 μg/dl), or cortisol (200, 400, 800 nmol/L) for 5 days. The supernatant was aspirated, and the MDMs were lysed. Supernatant was centrifuged to pellet the bacteria, and the pellets were added to the cell lysates. The bacterial suspensions were ultrasonically dispersed, serially diluted, and plated in triplicate on 7H10 agar. The number of resultant colonies was counted after 3 weeks. The *P* values were derived using an unpaired, 2-tailed, independent *t* test. The mean values and SD are shown for the number of CFUs per well.

**Figure 6:**
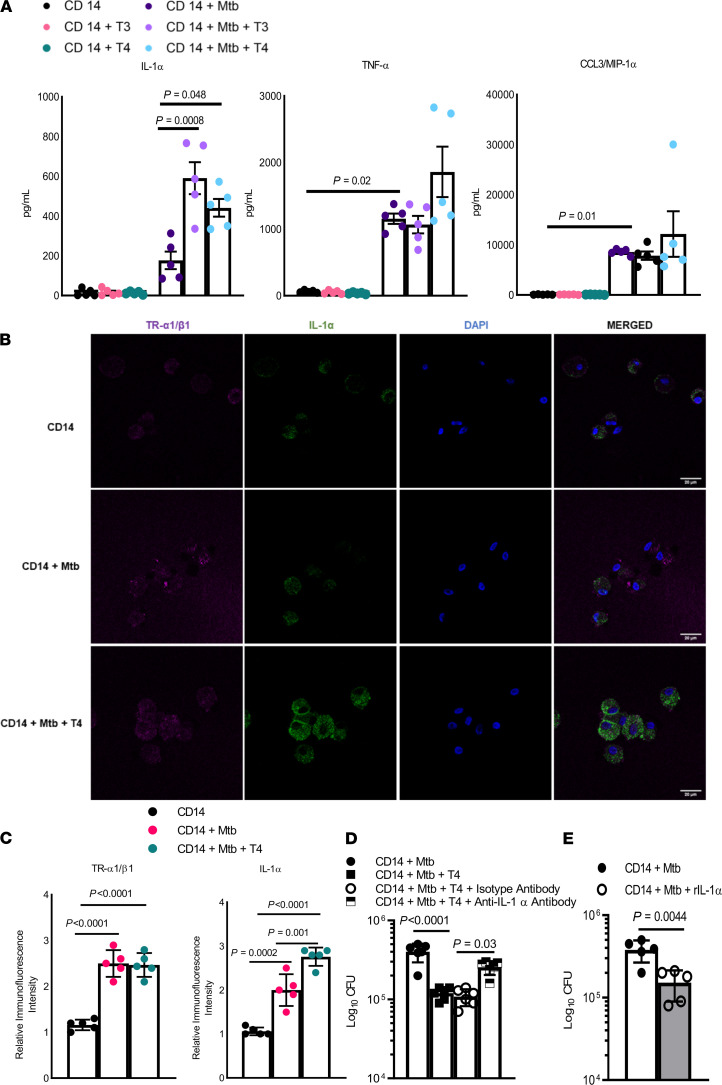
Thyroid hormones induce IL-1α production by Mtb-infected monocytes/monocyte-derived macrophages. Freshly isolated monocytes/monocyte-derived macrophages (MDMs) from 18- to 30-year-old donors (*n* = 5) were infected with H37Rv at an MOI of 2.5, as described in Methods. Some of the infected MDMs were cultured in the presence of T3 (15 nmol/L) or T4 (150 nmol/L) for 5 days. (**A**) The supernatants were aspirated, and cytokine and chemokine production were measured by multiplex ELISA. *P* values were derived using 1-way ANOVA with Tukey’s multiple-comparison test. The mean values, SD, and *P* values are shown. (**B**) The Mtb-infected MDMs in the presence or absence of T4 (150 nmol/L) were examined for TR-α1/β1 expression and IL-1α production by confocal microscopy. The images were taken at 63× magnification with oil immersion. Scale bar: 20 μm. (**C**) The respective immunofluorescence intensities are shown (*n* = 5). *P* values were derived using 1-way ANOVA with Tukey’s multiple-comparison test. Mean values, *P* values, and SD are shown. (**D**) Freshly prepared MDMs from 18- to 30-year-old donors (*n* = 6) were infected with H37Rv at an MOI of 2.5, as described in Methods. The infected MDMs were cultured in the presence of T4 (150 nmol/L), and the anti–IL-1α (10 μg/mL) antibody was added to some wells. The supernatant was aspirated; MDMs were lysed and plated on 7H10 agar. The number of colonies was counted after 3 weeks. The mean ± SD and *P* values are shown. The *P* values were determined using 1-way ANOVA with Tukey’s multiple-comparison test. (**E**) The MDMs (*n* = 5) were infected with H37Rv and cultured in the presence or absence of recombinant IL-1α (10 ng/mL); the number of bacterial colonies was determined. The *P* values were derived using an unpaired, 2-tailed, independent *t* test. Mean values and SD are shown.

*Monocytes/monocyte-derived macrophages upregulate thyroid hormone receptors in response toγ-Mtb stimulation*. We determined the expression of thyroid (TR1α and TR1β) and steroid hormone receptors (glucocorticoid receptor [GR1]) by various immune cell populations within freshly isolated PBMCs before and after stimulation with γ-irradiated Mtb (γ-Mtb). As shown in Figure 5, A and B and Supplemental Figure 8, TR1α, TR1β, and glucocorticoid receptor expression were significantly higher in monocytes than in NK, T, NKT, and B cells from fresh PBMCs. γ-Mtb significantly enhanced TR1α, TR1β, and glucocorticoid receptor expression by macrophages compared with NK cells, B cells, and T cells (Figure 5, A and B, and Supplemental Figures 6–8). In γ-Mtb–stimulated PBMCs, we also found higher expression of TR1α on NK and T cells compared with unstimulated PBMCs (Figure 5A). Further, Mtb H37Rv infection enhanced the expression of thyroid hormone receptor TRα1/β1 complex on human monocytes/monocyte-derived macrophages (MDMs) (*P* < 0.0001, Figure 6, B and C). Deiodinase (DIO) type 2 (D2) converts prohormone T4 into the active hormone T3 in macrophages and plays a crucial role in cytokine production and phagocytosis (39, 40). Control and Mtb H37Rv–infected human MDMs expressed DIO 1, 2, and 3, as determined by Western immunoblotting (Supplemental Figure 9).

### T3 and T4 hormones restrict Mtb growth in human MDMs.

We determined the effects of thyroid and steroid hormones on intracellular mycobacterial growth in MDMs. Freshly prepared MDMs from 7 healthy individuals aged 18–30 years were infected with Mtb H37Rv at an MOI of 2.5 and cultured in the presence or absence of various concentrations of the hormones T3, T4, cortisol, and DHEA. After 120 hours, 4.6 × 10^5^ ± 0.54 × 10^5^ CFUs per well of untreated MDMs were present. Addition of T3 (15 nmol/L) and T4 (150 nmol/L) hormones significantly reduced the number of CFUs in the MDMs (from 4.6 × 10^5^ ± 0.54 × 10^5^ to 6.7 × 10^4^ ± 0.21 × 10^4^, *P* < 0.0001, for T3 and 1.7 × 10^5^ ± 0.76 × 10^5^, *P* = 0.0006, for T4) (Figure 5C). However, supplementation with the cortisol and DHEA hormones had no effect on Mtb growth in MDMs (Figure 5C). The viability of the hormone-treated and untreated MDMs was similar (Supplemental Figure 10). Our results demonstrated that thyroid hormones inhibited Mtb growth in MDMs more efficiently than steroid hormones.

*Hormones enhance IL-1αproduction by Mtb-infected MDMs*. To determine the mechanism(s) by which T3 and T4 hormones inhibit Mtb growth in human MDMs, we measured various cytokine and chemokine levels in the culture supernatants of Mtb-infected MDMs by multiplex ELISA. Freshly prepared MDMs from 5 healthy individuals from the 18- to 30-year-old age group were infected with Mtb H37Rv at an MOI of 2.5 and cultured in the presence or absence of T3 (15 nmol/L) and T4 (150 nmol/L). Addition of T3 and T4 hormones to Mtb-infected MDMs enhanced the production of IL-1α (*P* = 0.0008 in the presence or absence of T3; *P* = 0.048 in the presence or absence of T4) (Figure 6A and Supplemental Figure 11). We also confirmed the above findings with confocal microscopy, and the H37Rv-infected human MDMs produced more IL-1α in the presence of the T4 hormone (Figure 6C).

*Hormones inhibit Mtb growth in human MDMs through IL-1αproduction*. Next, we determined whether T3 or T4 hormone–mediated IL-1α production restricts Mtb growth in human MDMs. Freshly prepared MDMs from 6 healthy individuals aged 18–30 years were infected with Mtb H37Rv and cultured in the presence of T4 hormone. Some of the T4 hormone–treated and untreated wells were treated with anti–IL-1α antibody. As shown in Figure 6D, after 120 hours, 4.1 × 10^5^ ± 1.07 × 10^5^ CFUs per well of untreated MDMs were present. Addition of T4 hormone reduced the number of CFUs to 1.2 × 10^5^ ± 0.20 × 10^5^ (*P* < 0.0001; Figure 6D). The addition of the anti–IL-1α antibody enhanced Mtb growth in T4 hormone–treated wells (1.2 × 10^5^ ± 1.07 × 10^5^ to 2.5 × 10^5^ ± 0.51 × 10^5^ CFU, *P* = 0.03; Figure 6D). The treatment of Mtb-infected MDMs with recombinant IL-1α also restricted Mtb growth (from 3.8 × 10^5^ ± 0.51 × 10^5^ to 1.5 × 10^5^ ± 0.72 × 10^5^ CFUs, *P* = 0.004; Figure 6E).

### Thra1^PV/+^ mice are susceptible to Mtb infection.

**Figure 7:**
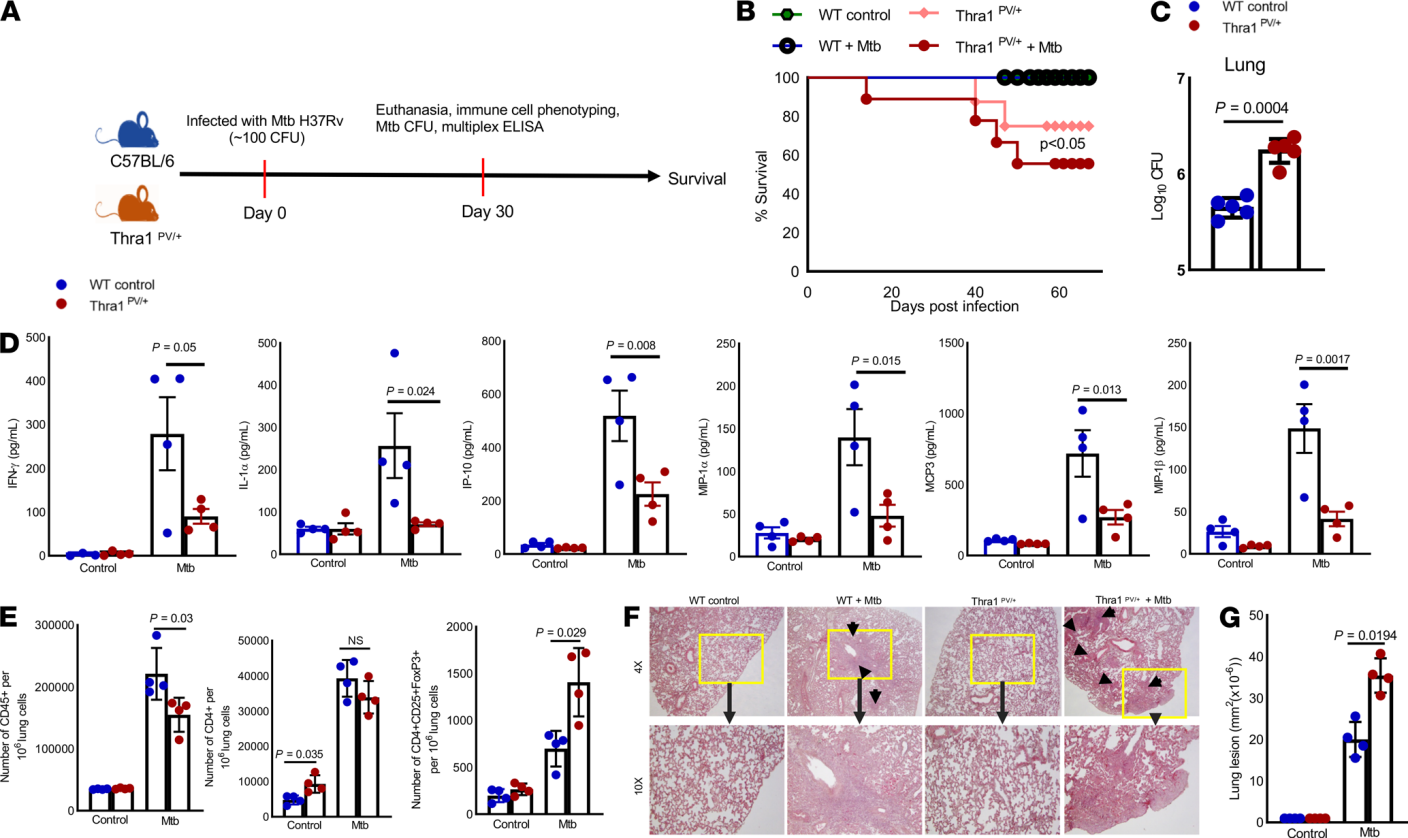
Thra1^PV/+^ mice are susceptible to Mtb infection. Six- to 8-week-old WT and Thra1^PV/+^ mice (both C57BL/6 background) (10 mice per group) were infected with approximately 100 CFUs of Mtb H37Rv by aerosol inhalation. (**A**) A schematic representation of the experiment is shown. (**B**) The *P* value for percentage survival was calculated using the log-rank test. The Kaplan-Meier survival curves of mice are shown. Data were pooled from 2 independent experiments (*n* = 5 mice in 2 experiments). (**C**) One month after infection, the bacterial burden in terms of CFUs in the lungs (*n* = 5 per group) was measured. Mean ± SD and *P* values are shown. The *P* values were derived using 1-way ANOVA with Tukey’s multiple-comparison test. (**D**) At 1 month after infection, lung homogenates from uninfected control and Thra1^PV/+^ mice and Mtb-infected control and Thra1^PV/+^ mice (*n* = 4) were collected, and cytokine and chemokine levels were measured with multiplex ELISA. (**E**) The numbers of CD45^+^, CD4^+^, and Tregs (CD4^+^CD25^+^FoxP3^+^) in various immune cell populations were measured by flow cytometry. Mean ± SD and *P* values are shown. *P* values were derived using 1-way ANOVA with Tukey’s multiple-comparison test. (**F**) At 1 month after infection, lungs from uninfected control and Thra1^PV/+^ mice and those from Mtb-infected control and Mtb-infected Thra1^PV/+^ mice were isolated and formalin-fixed. Paraffin-embedded tissue sections were prepared, and H&E staining was performed. Total original magnification power = 10 ocular x 4 objective = 40 and 10 ocular x 10 objective = 100.(**G**) Lung lesions were compared between groups. Data are representative of 2 independent experiments (*n* = 4 mice per group). *P* values were derived using 1-way ANOVA with Tukey’s multiple-comparison test. Data are expressed as the mean ± SD and *P* values are shown.

To determine the role of thyroid hormone signaling in Mtb infection, we infected WT and Thra1^PV/+^ mice expressing a dominant-negative thyroid receptor α1 mutant (TRα1PV) with Mtb H37Rv as described in the Methods (Supplemental Figure 12 and Figure 7A) (41). Thra1^PV/+^ mice were dwarfs, and by 6 weeks of age, the mean body weight of Thra1^PV/+^ mice was 12.4 ± 2.9 g, which was approximately 40% less than that of their WT littermates (Supplemental Figure 13) (41). Mtb infection significantly reduced the weight of Thra1^PV/+^ mice compared with that of uninfected WT or Thra1^PV/+^ mice (Supplemental Figure 13). As shown in Figure 7B, by 65 days after infection, 44.5% of the Mtb-infected Thra1^PV/+^ mice had died, whereas only 25% of the uninfected Thra1^PV+^ mice had died. In contrast, all WT-infected or uninfected mice survived (Figure 7B). One month after Mtb infection, there was a significant increase in the number of CFUs of bacteria in the lungs of Thra1^PV+^ mice compared with WT mice infected with Mtb (1.8 × 10^6^ ± 0.22 × 10^6^ vs. 0.45 × 10^6^ ± 0.047 × 10^6^ CFUs, *P* = 0.0004, Figure 7C).

### Mtb-infected Thra1^PV/+^ mice have defective immune responses.

We asked whether lack of thyroid hormone signaling had any effect on immune responses during Mtb infection. WT and Thra1^PV/+^ mice were infected with Mtb, and after 1 month the levels of various cytokines and chemokines were measured in lung homogenates by multiplex (34-plex) ELISA. As shown in Figure 7D and Supplemental Figure 14, levels of inflammatory cytokines IFN-γ and IL-1α and chemokines IP-10, MIP-1α, MCP-3, and MIP-1β were significantly lower in whole lung homogenates of Mtb-infected Thra1^PV/+^ mice than in homogenates of Mtb-infected WT mice (Figure 7D). We also examined various leukocyte populations by flow cytometry. The absolute numbers of total CD45^+^ and CD45^+^F4/80^+^ cells were significantly lower, whereas the numbers of Tregs (CD4^+^CD25^+^FoxP3) and CD45^+^CD11c^+^ cells were significantly higher at 1 month after Mtb infection in the lungs of Mtb-infected Thra1^PV/+^ mice than in Mtb-infected WT control or uninfected Thra1^PV/+^ mice (Figure 7E and Supplemental Figures 15 and 16). Histological analysis revealed that the number of lesions throughout the lungs was significantly higher in the lungs of Mtb-infected Thra1^PV/+^ mice than in Mtb-infected WT mice or uninfected Thra1^PV/+^ mice (Figure 7, F and G).

## Discussion

In the current study, we followed 839 healthy HHCs of TB patients for 2 years, obtained blood samples at 4-month intervals, and determined various factors correlated with the development of active TB in some of the HHCs. During the 24-month follow-up period, 17 HHCs developed active TB, and 14 (82.4%) of these progressors were in the 15- to 30-year age group. This suggests that the development of active TB in HHCs is age-dependent (relative risk, 3.75). At baseline (the “0” time point, when all the individuals were healthy), levels of circulating thyroid hormone T4 were significantly lower in the progressors compared with age-matched nonprogressors. At baseline and follow-up (a blood sample was obtained when active TB developed), freshly isolated PBMCs of the progressors had more CD16^+^CD56^+^ cells and Tregs than nonprogressors. At baseline, ESAT-6– and CFP-10–stimulated PBMCs of progressors produced more IFN-γ, IL-13, IL-10, and IL-18 and less IL-1α compared with ESAT-6– and CFP-10–stimulated PBMCs of nonprogressors. In the presence of thyroid hormones T3 and T4, monocytes significantly inhibited Mtb growth in human MDMs in an IL-1α–dependent manner. We also found that Thra1^PV/+^ mice (with defective thyroid hormone signaling) were more susceptible to Mtb infection than WT mice. Our studies identified additional risk factors for the development of active TB in the healthy HHCs of TB patients.

The HHCs of TB patients in developing countries are at an increased risk of development of active TB (25). It is known that this risk is further enhanced by HIV infection, alcoholism, smoking, diabetes, and other immunosuppressive conditions (42–45). However, there is limited information about the risk factors that enhance the development of active TB in healthy HHCs (10–21). Age is a major factor associated with Mtb infection (46). Children are at an increased risk of TB infection and disease progression because of their immature immune system (47, 48). Similarly, the geriatric population represents a large reservoir of LTBI and is at high risk of developing active disease because of a weak immune system (49, 50). In the current study, we found that adolescents could be at risk for development of TB and that there was a correlation between decreased circulating thyroid hormone T4 levels in the progressors compared with age-matched nonprogressors. Hormones can directly affect the activity of immune cells and control inflammatory, autoimmune, or infectious immune responses (51, 52). The bidirectional communication between the neuroendocrine and immune systems is well established (53). Glucocorticoids can inhibit Th1 responses, whereas their natural antagonist DHEA is able to promote such responses (54). TB patients have altered thyroid hormone and DHEA levels (55). In inbred rabbits, administration of T3 or T4 hormone markedly increased resistance to Mtb infection and hypothyroidism enhanced the susceptibility to Mtb infection (56). In the current study, HHCs who developed active TB disease during the follow-up period had a low level of serum T4 at baseline (Figure 4). TSH levels are regulated by T4 hormone through a negative feedback loop (57). However, our study found similar levels of TSH in nonprogressors and progressors at baseline (Supplemental Figure 4C). Our study demonstrated, to our knowledge, that decreased circulating T4 hormone levels at baseline can be a risk factor for young adult HHCs of TB patients.

At baseline, ESAT-6– and CFP-10–stimulated PBMCs of progressors produced less IL-1α than the stimulated PBMCs of the nonprogressors (Figure 3). IL-1α–deficient mice are susceptible to infection with low-dose aerosolized Mtb (58). Furthermore, in vivo neutralization of IL-1α renders mice highly susceptible to Mtb, and IL-1α is essential for the establishment of host resistance to Mtb. Chronic Mtb infection in IL-1α knockout mice causes large and diffuse inflammatory lesions in the lungs and the dispersed distribution of Mtb-infected cells (58). This suggests the important role of IL-1α–driven cell-cell crosstalk in coordinating protective granuloma formation or maintenance (58, 59). Our study demonstrated a correlation between decreased IL-1α production at baseline and the development of active TB disease at follow-up.

We found that monocytes are the major cell population that express thyroid and glucocorticoid receptors. T3 and T4 but not cortisol and DHEA enhanced IL-1α production by Mtb-infected macrophages and inhibited Mtb growth in an IL-1α–dependent manner. Thyroid receptor-α expression on macrophages modulates the inflammation caused by infection (60). Thyroid hormone–treated macrophages display the M1 phenotype and show improved capacity for phagocytosis (60). We have not determined the IL-1α–dependent mechanisms that inhibit Mtb growth in macrophages. However, it is known that IL-1 signaling inhibits Mtb growth in a COX2-dependent manner by suppressing IFN-1 production (58). In the current study, we found that young HHCs with reduced circulating T4 levels develop active TB and found a mechanism by which the T3 and T4 hormones inhibited Mtb growth in an IL-1α–dependent manner.

In addition to the above findings, we also found higher numbers of CD16^+^CD56^+^ cells and Tregs in fresh PBMCs; increased IFN-γ, IL-13, and IL-10 production; and reduced IL-1α production by the ESAT-6– and CFP-10–stimulated PBMCs of progressors compared with nonprogressors at baseline. NK cells are an important component of the innate immune response and kill Mtb-infected cells (61, 62). CD16 (Fc receptor γRIIIa) is a mediator of antibody-dependent cell-mediated cytotoxicity and is expressed by a subpopulation of NK cells (63). Antibody recognition by CD16 results in the activation of NK cells, release of cytolytic granules, and the killing of target cells (63). In the current study, we found that CD16-expressing NK cell numbers were high in the progressors at baseline compared with nonprogressors, and this number was reduced when HHCs developed active TB disease. We have not determined the function of CD16^+^ NK cells in progressors and nonprogressors, but our study results are in accordance with those of a recent cohort study that found lower numbers of peripheral blood NK cells in patients with active TB, whereas individuals with LTBI had a normal NK cell number. Recent studies in nonhuman primates demonstrated that CD27^+^ NK cells accumulate more in the lung of LTBI-positive macaques compared with macaques with active TB (64). This NK cell population was also detected in circulation of LTBI-positive individuals and expanded in response to Mtb antigens (33).

At baseline and follow-up (at the time of the development of active disease), we found higher numbers of Tregs in the PBMCs of the progressors compared with those in the PBMCs of the nonprogressors. Activated CD4^+^ T cells can transiently express CD25 and FoxP3 (65), and we have not measured the suppressive function of CD4^+^CD25^+^FoxP3^+^ cells. It is possible that increased numbers of CD4^+^CD25^+^FoxP3^+^ cells in the PBMCs of the progressors may be due to increased activated CD4^+^ cells. ESAT-6– and CFP-10–stimulated PBMCs of progressors produced more IL-10 than the stimulated PBMCs of nonprogressors at baseline and at follow-up. Tregs produce IL-10 and play an important role in modulating Mtb-specific immune responses (66). Tregs in lymph nodes reduce the frequency of Mtb-responsive IFN-γ–producing CD4^+^ and CD8^+^ T cells, and there is an association between the number of Tregs and the severity of Mtb infection (31, 67). The higher preinfection frequencies of Treg cells in Mtb-infected cynomolgus macaques was associated with a higher likelihood of latent TB (68). Our study suggests that the increased numbers of Tregs along with increases in other markers at baseline in young HHCs of TB patients can serve as a biomarker for the future development of active TB.

We found that the Mtb antigen–stimulated PBMCs of the progressors produced more IFN-γ and IL-13 than the stimulated PBMCs of the nonprogressors at baseline. In contrast, the ESAT-6– and CFP-10–stimulated PBMCs of progressors produced less IFN-γ, IL-17, and IL-1α compared with the stimulated PBMCs of the nonprogressors at follow-up (when the progressors developed active TB). The essential protective role of these cytokines during Mtb infection is well known (69). The higher numbers of CD56^+^CD16^+^ cells and higher levels of protective cytokine production at baseline in the progressors suggest that strong host defense mechanisms are in place to protect the progressors during the early stages of infection, but the failure of these mechanisms leads to the development of active TB disease. This failure was demonstrated by reduced numbers of CD56^+^CD16^+^ cells and the production of associated cytokines in progressors.

Similar to the above findings in humans, Mtb-infected Thra1^PV/+^ mice demonstrated defective effector cytokine (IL-1α and IFN-γ) and chemokine (IP-10, MIP-1α, MIP-1β, and MCP-3) production, a higher number of Tregs, a higher bacterial burden in the lungs, and higher mortality than Mtb-infected WT mice (45.4% of the mice died within 65 days, Figure 7). Thyroid hormone signaling can affect immune cell functions, such as chemotaxis, phagocytosis, ROS generation, cytokine production, and metabolism (70). Given that T4 and T3 play pleiotropic roles in metabolism, our findings with Thra1^PV/+^ mice may not be specific for Mtb infection and it is possible that Thra1^PV/+^ mice may be susceptible to other infections. Our human and mouse findings suggest that thyroid hormone signaling is essential for the generation of optimal immune responses during Mtb infection.

In summary, we followed a large number of healthy HHCs of TB patients, obtained blood samples at regular intervals, and identified the risk factors that enhanced the development of active TB. Our findings suggest that young HHCs of TB patients with increased numbers of CD16^+^CD56^+^ cells and Tregs as well as decreased production of T4 hormone and IL-1α at baseline are at an increased risk of developing active TB disease.

## Methods

### Human studies

#### Cohort description.

Over 4 years, a total of 839 household contacts were enrolled in the study. The following were used as the enrollment criteria.

Index patients were newly diagnosed, sputum-positive pulmonary TB patients attending the Designated Microscopy Centers under TB clinics at Bhagwan Mahavir Medical Research Centre (BMMRC) and Blue Peter Public Health & Research Centre (BPHRC) in Hyderabad, India. Active TB diagnosis was performed by following the Revised National Tuberculosis Control Programme (RNTCP) (now National Tuberculosis Elimination Programme, NTEP) guidelines.

Household members residing in the same house as the index case for a minimum of 3 months prior to the date of diagnosis of TB were identified. These individuals had shared at least 5 meals per week with the index case and had no past history of TB or ATT. The demographic details of the HHCs, including age, sex, history of Bacillus-Calmette-Guerin (BCG) vaccination, history of pulmonary TB, and smoking and drinking profiles, were collected and are shown in Table 1.

The exclusion criteria were HHCs having HIV, diabetes, autoimmune diseases, or any other immunosuppressive condition; children younger than 15 years were excluded. If participants developed comorbid conditions and started using tobacco (chewing tobacco or smoking) or alcohol (more than 2 drinks per week) during the study, their data were excluded from the final analysis.

#### Initial screening of the participants.

Prior to enrollment in the study, HHCs were screened to ensure that they meet the eligibility criteria outlined above. Screening evaluations included eligibility assessment, informed consent, detailed medical history, and exposure assessment to the index case. Local laboratory evaluations to rule out HIV, diabetes, and any other chronic conditions were performed. After enrollment, baseline visits were scheduled and blood samples were collected. Demographics and data on smoking and alcohol usage were collected. Physical examination was done and participants’ height and weight were noted. Blood samples were used to perform immune assays and hematological analysis. Participants were categorized as having LTBI by an in-house assay as outlined in the section below.

#### Latent TB in the study cohort.

HHCs were categorized as LTBI-positive or LTBI-negative using an in-house interferon gamma release assay (IGRA) (Supplemental Data Set 1) (71). The test was performed every 4 months irrespective of baseline IGRA result. The HHCs with symptoms consistent with TB were evaluated by chest radiography and clinical evaluation. A complete blood panel was performed to study the hematological correlates of the progression to TB as outlined in Supplemental Figure 1.

#### Follow-up of HHCs.

All HHCs were evaluated every 4 months for 2 years. At each visit, they were evaluated for TB, and 20 mL of blood was collected. Blood samples were used to perform immune assays and hematological analysis. An in-house assay was performed to identify LTBI converters and nonconverters (71). All participants with symptoms of possible TB were referred for clinical evaluation and treatment.

#### Outcome determination.

The outcomes of the study were as follows: 1) completion of the 2-year follow-up; 2) development of TB in some HHCs; and/or 3) withdrawal of the participants from the study.

Progressors were classified as those with signs and symptoms consistent with active TB and those with 1) clinical specimens that showed acid-fast bacilli in the test smear or Mtb in culture or 2) chest radiographic changes consistent with TB and a clinical response to ATT. The nonprogressors were individuals who completed their follow-up without development of TB.

#### TB treatment in active TB progressors.

Seventeen of the HHCs progressed to active TB during the follow-up. These HHCs were referred to the RNTCP (now NTEP) centers for treatment. All the TB progressors were screened for rifampicin resistance following RNTCP guidelines, and no drug resistance was observed in any of them. Standard fixed-dose combination (FDC) first-line ATT was administered to the patients according to their weight bands. Treatment was administered daily for a minimum of 6 months with 4 standard FDC drugs in the intensive phase and 3 drugs during the continuation phase. Although TB activation was an off-study criterion in this study, samples were collected from progressors after TB treatment for hormone assays.

#### Determination of LTBI in the HHCs.

LTBI in the HHCs was determined according to our previously published protocols (71). A total of 2 *×* 10^6^ human PBMCs were stimulated with and without 10 μg/mL CFP-10 and ESAT-6 antigens and incubated at 37°C for 96 hours. IFN-γ released by PBMCs was measured with a sandwich ELISA using a commercial human IFN-γ kit (eBioscience Inc.) by following the manufacturer’s instructions. The IFN-γ concentration was calculated using MPM software version 6.1. HHCs were categorized as either LTBI-negative or LTBI-positive depending on the IFN-γ value.

#### Complete blood count analysis.

Whole blood collected in EDTA was subjected to complete blood count analysis by using an automated cell counter (Mindray Biochemistry Analyzer, Golden Harvest Industries). A total of 200 μL of the whole blood collected in an EDTA tube was used for the complete blood count analysis. The WBC, RBC, and platelet counts were determined by impedance method, and hemoglobin levels were determined by colorimetry.

#### Antibodies and other reagents.

PE anti-CD16 (catalog 555407), PerCP anti-CD14 (catalog 340585), and APC anti-CD56 (catalog 555518) were used to identify monocytes and NK cells. FITC anti-CCR7 (catalog 561271), PE anti-CD27 (catalog 555441), PerCP anti-CD3 (catalog 555332), and APC anti-CD56 (catalog 555518) were used to identify the T cell and NK cell populations. FITC anti-CD4 (catalog 555346), PE anti-Foxp3 (catalog 560046), and APC anti-CD25 (catalog 555434) were used to label Tregs (all antibodies used were obtained from BD Biosciences). To determine the expression of thyroid hormone receptors, we used Alexa Fluor 488 anti–thyroid hormone receptor β and FITC-thyroid hormone receptor alpha (Bioss Antibodies) and performed intracellular staining using an intracellular staining kit (BioLegend). In addition, we used PE anti–human GPR83 (BioLegend) to determine the expression of glucocorticoid receptors.

#### Isolation and culture of PBMCs.

PBMCs were isolated by density gradient centrifugation using Ficoll-Hypaque. Whole blood diluted with RPMI was layered over an equal volume of Ficoll and centrifuged for 30–40 minutes at 2000 RPM without braking. PBMCs were washed twice and counted by trypan blue staining. Freshly isolated PBMCs were cultured in 24-well plates at 1 × 10^6^ cells/well in RPMI 1640 containing 1% penicillin/streptomycin (Sigma-Aldrich), L-glutamine, and 10% heat-inactivated human serum with or without CFP-10 + ESAT-6 (10 μg/mL) at 37°C in a humidified atmosphere with 5% CO_2_. After 96 hours, cell-free culture supernatants were collected, aliquoted, and stored at –70°C until the cytokine concentrations were measured by ELISA according to the manufacturer’s guidelines.

#### Antigens for stimulation assays.

For stimulation of PBMCs, we used ESAT-6 and CFP-10 peptide pools (BEI Resources), consisting of 21 and 22 peptides covering the entire 6 kDa ESAT-6 and 10 kDa CFP-10 protein sequence, respectively. A total of 1 *×* 10^6^ human PBMCs were stimulated with and without 10 μg/mL CFP-10 and ESAT-6 antigen and incubated at 37°C for 96 hours. For some experiments, freshly isolated PBMCs were cultured with or without γ-Mtb H37Rv (BEI Resources) (10 μg/mL) for 120 hours.

#### Flow cytometry.

Briefly, a total of 1 × 10^6^ PBMCs were stained for 3 cell subsets. T cells, monocytes, and the NK cell subsets were surface stained with respective antibodies and incubated in the dark for 30 minutes before being analyzed by flow cytometry. To determine the FoxP3 population, we used an intracellular staining kit from BioLegend. For surface staining, CD4 and CD25 were added to the cells before they were permeabilized. Anti-FoxP3 antibody was then added to the cells resuspended in staining buffer. After incubation, the cells were washed twice and fixed in 1% paraformaldehyde before acquisition on a FACSCalibur (BD Biosciences).

#### Gating strategies.

CD4^+^ cells were identified after gating on the whole lymphocyte population. Tregs were gated as CD4^+^CD25^+^FoxP3^+^ cells. CD16-expressing CD56^+^ cells were identified as double-positive CD16^+^CD56^+^ cells. CD16-expressing monocytes were identified as CD14^+^CD16^+^ cells.

#### Measurement of T3, T4, DHEA, and cortisol.

T3, T4, DHEA, and cortisol concentrations in the serum were measured by ELISA by following the manufacturer’s instructions (T3 and T4, Meril Diagnostics) (DHEA and cortisone, Diagnostics Biochem Canada). Briefly, 25 μL of standards (positive controls provided by the manufacturer for the purpose of assay) or patient sera were added to the respective precoated wells. Fifty microliters of biotin and 100 μL of the enzyme conjugate were added to all the wells. The samples were incubated for 60 minutes at room temperature (RT). The wells were washed, and 100 μL of the substrate was added. The stop solution was added after 15–20 minutes of incubation in the dark. The absorbance was read at 450 nm. The samples were run in batches, and assays were performed by commercial vendors who were blinded to the specimen status.

#### Measurement of cytokine production.

ESAT-6– and CFP-10–stimulated PBMCs as well as the T3-, T4-, DHEA-, and cortisol-treated H37Rv-infected MDM culture supernatants were stored at –70°C until the cytokine concentrations were measured. In culture supernatants, the following 34 cytokines and chemokines were measured using a multiplex ELISA kit per the manufacturer’s instructions (34-Plex Human ProcartaPlex Panel 1A, Thermo Fisher Scientific): Eotaxin/CCL11; GM-CSF; GRO-α/CXCL1; IFN-α; IFN-γ; IL-1β; IL-1α; IL-1RA; IL-2; IL-4; IL-5; IL-6; IL-7; IL-8/CXCL8; IL-9; IL-10; IL-12 p70; IL-13; IL-15; IL-17A; IL-18; IL-21; IL-22; IL-23; IL-27; IL-31; IP-10/CXCL10; MCP-1/CCL2; MIP-1α/CCL3; MIP-1β/CCL4; RANTES/CCL5; SDF1-α/CXCL12; TNF-α; and TNF-β/LTA. The assay was run in batches by an independent reader blinded to the specimen status.

#### Determination of Mtb H37Rv growth in MDMs.

Monocytes were isolated from the PBMCs of healthy donors by magnetic beads conjugated to anti-CD14 (Miltenyi Biotec). CD14^+^ monocytes (0.5 × 10^6^ cells/well) were plated in 24-well plates in 1 mL of antibiotic-free macrophage serum-free medium (SFM) (Gibco) and incubated at 37°C in a humidified atmosphere in 5% CO_2_ for up to 6 days. The culture medium was renewed every day, and the cells were monitored morphologically for differentiation. After 6 days (after differentiation of the monocytes into macrophages), the cells were washed and infected with Mtb H37Rv at an MOI of 2.5 (1 MDM to 2.5 Mtb), incubated at 37°C for 2 hours, washed to remove the extracellular bacilli, and cultured in macrophage-SFM (Gibco). T3 (5, 10, and 15 nmol/L), T4 (50, 100, and 150 nmol/L), DHEA (200, 400, and 800 μg/dl), and cortisol (200, 400, and 800 nmol/L) were added to some of the wells. Anti–IL-1α (10 ng/mL) was added to some of the wells. The cells were cultured for 120 hours. The supernatant was aspirated, and MDMs were lysed. The supernatant was centrifuged to pellet the bacteria, and the pellets were added to the cell lysates. The bacterial suspensions were ultrasonically dispersed, serially diluted, and plated in triplicate on 7H10 agar. The number of colonies was counted after 3 weeks.

#### Confocal microscopy.

Confocal microscopy was performed to determine IL-1α and TR-1 α/β expression on control or Mtb-infected monocytes in the presence or absence of T4 hormone. The CD14^+^ monocytes (0.5 × 10^6^ cells/well) were plated in 24-well plates in 1 mL of antibiotic-free macrophage-SFM (Gibco) and incubated at 37°C in a humidified atmosphere with 5% CO_2_. After 2 hours, the cells were washed and infected with Mtb H37Rv at an MOI of 2.5 (1 MDM to 2.5 Mtb), incubated at 37°C for 2 hours, washed to remove the extracellular bacilli, and cultured in macrophage-SFM. T4 hormone was added to a few wells at a concentration of 150 nmol/L. For immunostaining, the cells were first washed briefly with PBS and fixed with 4% paraformaldehyde for 15–20 minutes at RT. The samples were then washed 3 times for 5 minutes with PBST. Fixed cells were then incubated with 0.025% Triton X-100 in PBS (PBST) for 10 minutes and subjected to 3 subsequent washes in PBST. Nonspecific binding was blocked by incubating the samples with 5% BSA in PBST for 1 hour and then washing 2 times for 5 minutes in PBST. The cells were then incubated at 4°C overnight in PBST with appropriate dilutions of the primary antibodies IL-1α (1:250) and TR-1 α/β (1:250), and subsequently the cells were washed thoroughly for 3 times for 5 minutes in PBST. Next, the cells were stained with their respective secondary antibodies at 1:1000 dilutions (v/v), washed again with PBST for 3 times for 5 minutes, and mounted using FluoroShield mounting medium with DAPI. The mounted slides were then examined under a Zeiss LSM 510 meta laser scanning confocal microscope. ZEN 2009 software (Zeiss) was used for 3–5 image acquisitions per sample; then, the images were processed and uniformly quantified for each experiment using ImageJ software (NIH). Representative images are shown from 5 independent experiments.

#### Western blot.

CD14^+^ monocytes were isolated from PBMCs of healthy volunteers and cultured at 1 × 10^6^ cells in 12-well plates to obtain MDMs for 2–3 days. MDMs were infected with Mtb H37Rv, and protein lysates were collected after 72 hours of infection using M-PER (mammalian protein extraction reagent) with protease and phosphatase inhibitor cocktail (Thermo Fisher Scientific). Total protein concentrations were estimated using the bicinchoninic acid method, and 15 μg of protein samples were used to perform Western blotting. Resolved protein gels were electroblotted onto a PVDF membrane and blocked with 5% nonfat dry milk in TBST buffer for 1 hour at RT. Then, the membranes were probed with Rb-DIO1 (Invitrogen, PA5-100139; 1:1000), goat-DIO2 (Novus, NBP1-00178; 1:1000), Rb-DIO3 (Novus, NBP1-05767; 1:1000), and Rb-GAPDH (Cell Signaling Technology, 2118; 1:1000) overnight at 4°C and followed by anti-rabbit (Cell Signaling Technology, 7074; 1:10000) or anti-goat (Santa Cruz Biotechnology, sc-2354; 1:3000) HRP-linked secondary antibody for 1 hour at RT. Protein bands were detected using the enhanced chemiluminescence method using Bio-Rad ChemiDoc imaging system.

#### Statistical analysis used for human studies.

Prism 7 software (GraphPad) was used for statistical analyses. Descriptive analyses were performed for all relevant variables prior to their inclusion in analyses. Frequency counts and percentages were obtained for interval and ordinal categorical data. For continuous variables, the central tendency (mean, mode, and median) and dispersion (range, variance, standard deviation, standard error, and coefficient of variation) were calculated. Possible outliers and/or influential observations were identified, and their validity was double-checked using available records. *P* values that were less than 0.05 were considered statistically significant. The results are expressed as the mean ± SD. For data that were normally distributed, comparisons between groups were assessed with ANOVA and 1-way repeated-measures ANOVA. When the overall ANOVA *P* values were statistically significant and post hoc tests were performed (*t* tests), *P* values were adjusted for multiple comparisons using Tukey’s multiple-comparison test. For data that were not normally distributed, Wilcoxon’s rank-sum test was used. The biomarker analysis program was used in MetaboAnalyst software to generate the ROC curves, and the AUC was determined for various hormones to predict the true TB progressors at baseline.

#### Human study approval.

From June 2014 through June 2018, we enrolled 839 HHCs (Figure 1) aged 15–73 years after obtaining written informed consent. The study was approved by the institutional ethics committees from BMMRC and BPHRC.

### Mouse studies

#### Animals.

Specific pathogen–free 4- to 8-week-old male and female WT C57BL/6 mice were obtained from The Jackson Laboratory, and Thra1^PV/+^ (C57BL/6 background) mice were provided in-house. Thra1^PV/+^ founder mice were back-crossed into the C57BL/6 background and maintained at the animal facility in the University of Texas Health Science Center at Tyler. Thra1^PV/+^ mice and littermates were genotyped (primers are listed in Supplemental Table 4) using the REDExtract-N-Amp Tissue PCR kit (MilliporeSigma) to confirm the presence of the approximately 586-bp PCR product.

#### Aerosol infection of mice with Mtb H37Rv.

Before infecting the mice with Mtb H37Rv, bacteria were grown in liquid medium until the mid-log phase was reached and then frozen in aliquots at –70°C. Bacterial counts were determined by plating on 7H10 agar supplemented with oleic albumin dextrose catalase (OADC). For infection, bacterial stocks were diluted in 10 mL of normal saline (0.5 × 10^6^ CFU/mL, 1 × 10^6^ CFU/mL, 2 × 10^6^ CFU/mL, and 4 × 10^6^ CFU/mL) and placed in a nebulizer within an aerosol exposure chamber that was custom-made by the University of Wisconsin. In preliminary studies, groups of 3 mice were exposed to the aerosol at each concentration for 15 minutes. After 24 hours, the mice were euthanized, and homogenized lungs were plated on 7H10 agar plates supplemented with OADC. CFUs were counted after 14–22 days of incubation at 37°C. The concentration that deposited approximately 75–100 bacteria in the lung during aerosol infection was used for further studies (Supplemental Table 5).

#### Lung cell preparation.

Lungs were harvested from the control and Mtb-infected WT C57BL/6 and Thra1^PV/+^ mice 30 days after Mtb challenge and were placed into 60 mm dishes containing 2 mL of HBSS. The tissues were minced with scissors into pieces no larger than 2–3 mm, and the fluid was discharged onto a 70 μm filter (BD Biosciences) that had been prewetted with 1 mL of PBS containing 0.5% BSA (Sigma-Aldrich) suspended over a 50-mL conical tube. The syringe plunger was then used to gently disrupt the lung tissue before washing the filter with 2 mL of cold PBS/0.5% BSA. The total number of viable cells in the lungs was determined with the trypan blue exclusion method. For flow cytometry experiments, we gated the total lung CD45^+^ cells (leukocytes) and measured various cell populations.

#### Flow cytometry and intracellular staining.

After the mice were euthanized, lungs were perfused with 5 mL of PBS via the left ventricle. Lungs were mechanically homogenized and passed through a 70 μm cell strainer. Remaining RBCs were lysed using BD Pharm Lyse (BD Biosciences). Cells were treated with an Fc blocker (TruStain, BioLegend) prior to staining. Surface staining to identify leukocyte populations was then performed. For FoxP3 intracellular staining, cells were permeabilized with 0.1% saponin and stained for intracellular FoxP3. The cells were washed, resuspended in FACS buffer, and analyzed by using an Attune flow cytometer.

#### Multiplex ELISA.

Mouse multiplex ELISA kits (36-Plex kits, Thermo Fisher Scientific) were used to measure chemokine and cytokine levels according to the manufacturer’s instructions.

#### Histopathology of necrotic lesions in the lung.

At the specified time points, mice were euthanized, and the harvested lungs were placed in 10% neutral buffered formalin (StatLab) for 48 hours to inactivate the infectious agent. Paraffin-embedded blocks were cut into 5 μm thick sections. For morphometric lesion analyses, the lung sections were stained with H&E and examined in a blinded manner to assess the necrotic lesions as previously described (72). Briefly, each lung lobe was quantified for the lesion area and percentage of lung lesions by using ImageJ (NIH). Two investigators, DT and RR, independently assessed the immunohistochemical readouts using morphometric analyses.

#### Statistics used for mouse studies.

Prism 7 (GraphPad) was used for statistical analyses. The results are shown as the mean ± SEM. Comparisons between groups were performed by a paired or unpaired *t* test, as appropriate. Mouse survival was compared using the Kaplan-Meier log-rank test.

#### Mouse study approval.

The IACUC of the University of Texas Health Science Center approved all the animal studies. Animal procedures involving the care and use of mice were performed in accordance with the guidelines of the NIH/OLAW (Office of Laboratory Animal Welfare).

## Author contributions

RV and VLV devised the project and the main conceptual ideas, proofed the outline, and conceived and planned the experiments. KPD, DT, and VSKN planned and carried out the experiments, performed the analysis, and designed the figures. PP and RKR carried out the experiments. KPS, DT, and KPD performed data analysis. SYC and SP provided Thra 1 PV^/+^ mice. MSA screened the patients and was involved in participant counseling, recruitment, and follow-up. MJ, RTNM, MGN, and SYC reviewed and edited the manuscript. DT, RV, and KPD drafted the manuscript. The order of co-senior authorship was based on the contribution of main conceptual idea, experimental designing, and drafting of manuscript.

## Supplementary Material

Supplemental data

Supplemental Data Set 1

## Figures and Tables

**Table 1:**
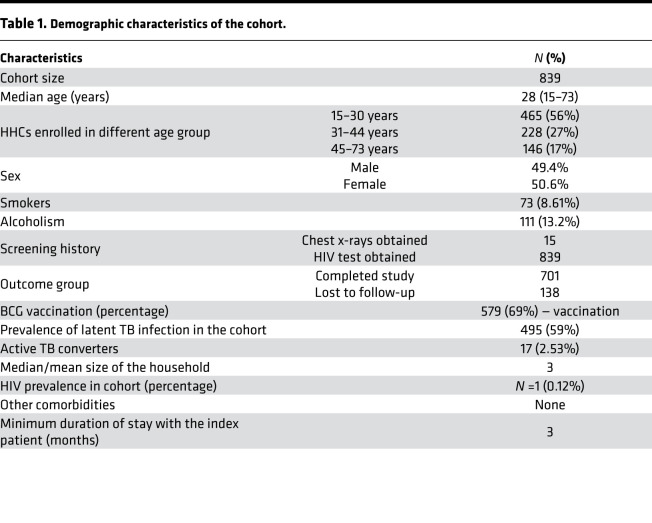
Demographic characteristics of the cohort.

## Version 1 07/08/2021

Electronic publication
