# Supplementary material for: Reduced thyroxine production in young household contacts of tuberculosis patients increases active tuberculosis disease risk
Source: JCI Insight. 2021 Jul 8;6(13):e148271. doi: 10.1172/jci.insight.148271 (PMC8410087; doi:10.1172/jci.insight.148271)
Supplement: Supplemental data [file jciinsight-6-148271-s148.pdf]

SUPPLEMENTARY MATERIAL

Reduced thyroxine production in young household contacts of tuberculosis patients increase active tuberculosis disease risk

Kamakshi Prudhula Devalraju[1,†], Deepak Tripathi[2,†], Venkata Sanjeev Kumar Neela[1,†], Padmaja Paidipally[2], Rajesh Kumar Radhakrishnan[2], Karan P. Singh[3], Mohammad Soheb Ansari[1], Martin Jaeger[4,5], Romana T. Netea-Maier[4,], Mihai G. Netea[5], Sunmi Park[6], Sheue-yann Cheng[6], Vijaya Lakshmi Valluri[1,$,*], Ramakrishna Vankayalapati[2,$,*]

†Equal contribution

[$]Shared Senior authorship

[1]Immunology and Molecular Biology Department, Bhagwan Mahavir Medical Research Centre, Hyderabad, Telangana, 500004, India

[2]Department of Pulmonary Immunology, Center for Biomedical Research, The University of Texas Health Center, Tyler, Texas, TX 75708, United States of America

[3]Department of Epidemiology and Biostatistics, School of Community and Rural Health, University of Texas Health Science Center, 11937 US Hwy 271, Tyler, TX 75708, United States of America

[4]Department of Internal Medicine, Division of Endocrinology, Radboud University Medical Center, Geert Grooteplein Zuid 8, 6525 GA, Nijmegen, The Netherlands

[5]Department of Internal Medicine and Radboud Center for Infectious Diseases, Radboud University Medical Center, Geert Grooteplein Zuid 8, 6525 GA, Nijmegen, The Netherlands
[6]Laboratory of Molecular Biology, National Cancer Institute, National Institutes of Health, Bethesda, MD 20892

***CORRESPONDENCE**

Ramakrishna Vankayalapati, Department of Pulmonary Immunology, University of Texas Health Science Center at Tyler, 11937 US Highway 271, Tyler, TX 75708-3154. Telephone (903) 877-5190, Fax (903) 877-7989. E-mail: krishna.vankayalapati@uthct.edu

OR

Vijaya Lakshmi Valluri, Immunology and Molecular Biology Department, Bhagwan Mahavir Medical Research Centre, Hyderabad, 500004, India. Telephone: 9140 23497306 E-mail: vijayavalluri@gmail.com

SUPPLEMENTAL INFORMATION

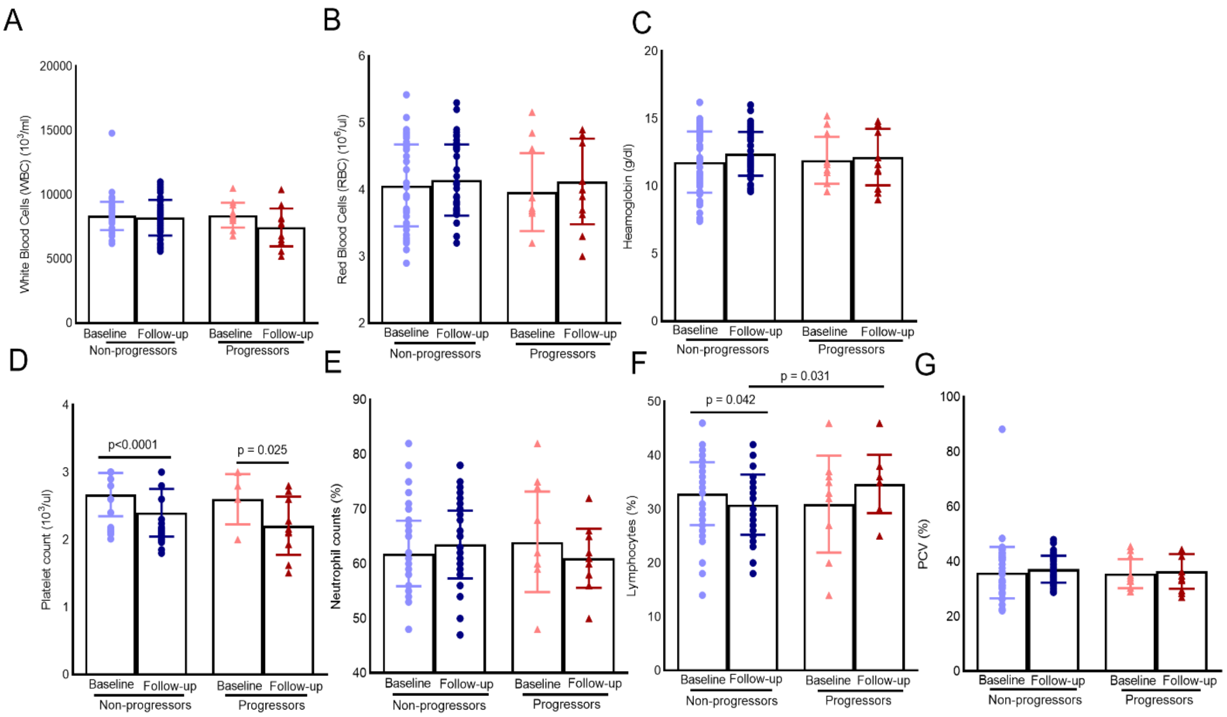

Figure S1. Complete blood profile of the HHCs. The components of the whole blood samples were assessed by automated cell counter as follows: (A) white blood cells, (B) red blood cells, (C) hemoglobin, (D) platelet count (E), neutrophil count, (F) lymphocytes and (G) PCV in non-TB progressors (n = 68) and TB progressors (n = 12) within the HHCs at baseline and follow-up (when the progressors were registered as having active TB). The p values were determined using one-way ANOVA with Tukey's multiple comparisons test. The mean values, SD, and p values are shown.

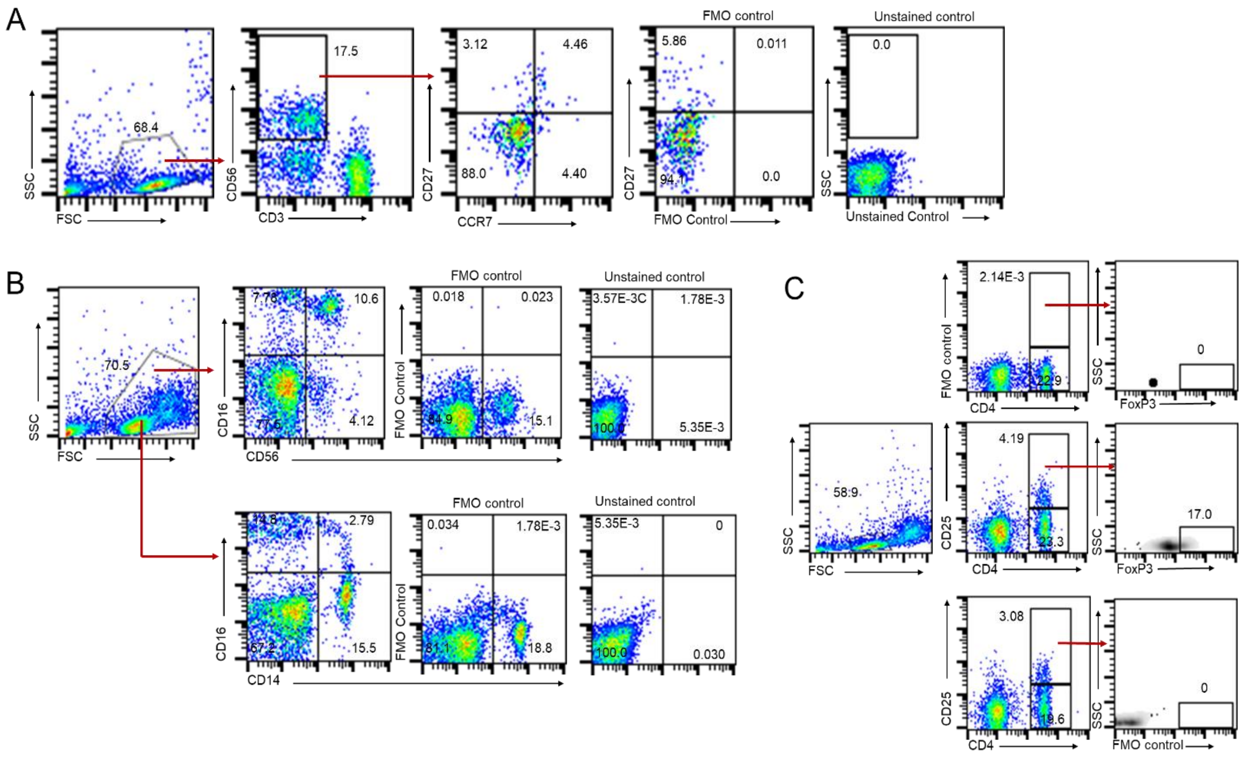

Figure S2. A representative flow cytometry figure showing the gating strategies. A representative flow cytometry figure for (A) CD3-CD56+CD27+CCR7+, (B) CD16+CD56+ and CD14+CD16+ (C) CD4+CD25+FoxP3+ cells is shown. This is a representative flow cytometry plots for Figure 2.

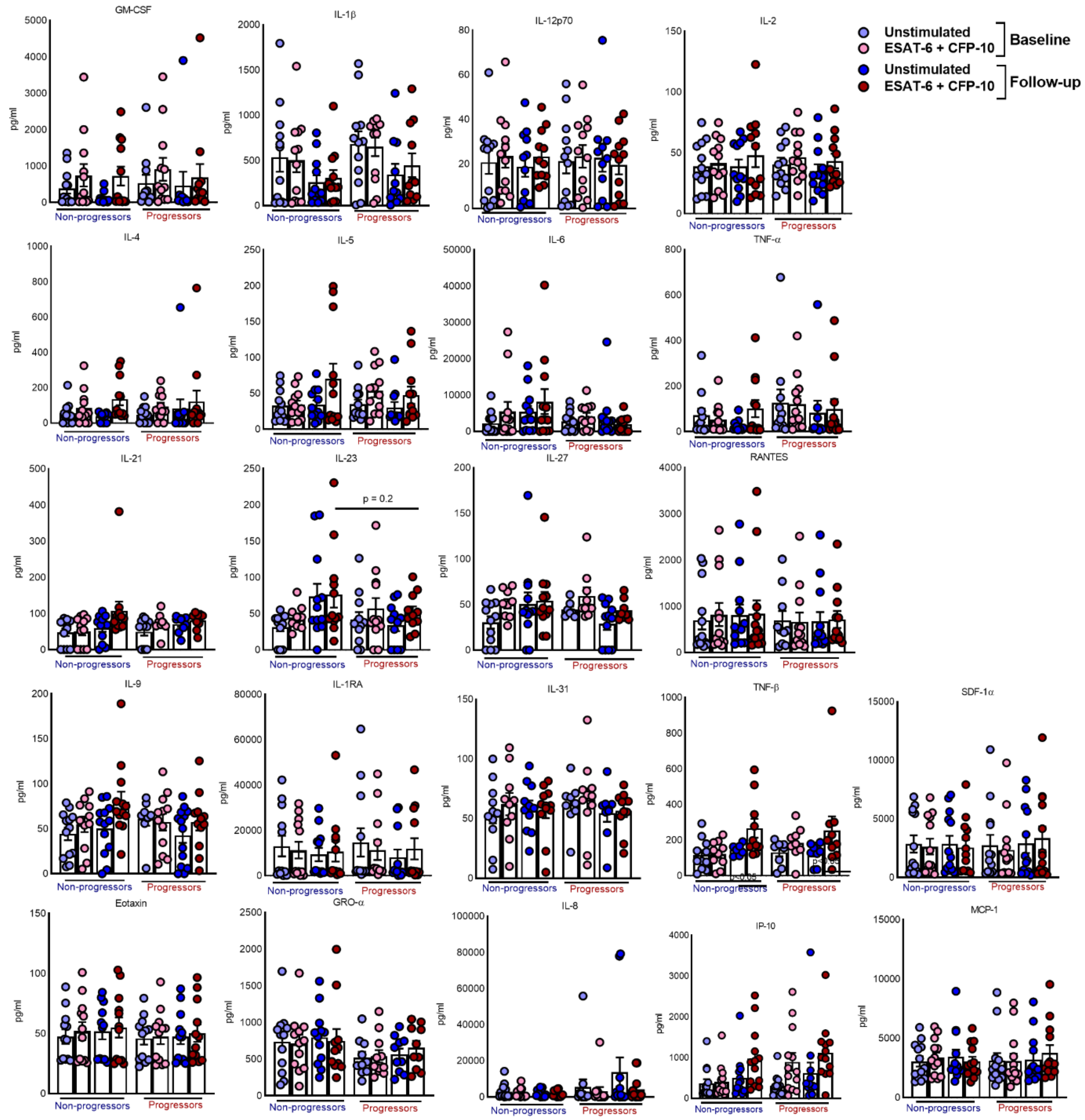

Figure S3. Cytokine and chemokine profiles of ESAT-6- and CFP-10-stimulated PBMCs. The PBMCs from the nonprogressors (n =12) and progressors (n = 12) at baseline and follow-up were isolated and cultured with or without ESAT6 and CFP10 (10 μg/ml each), as described in the methods section. After 96 hours, the culture supernatants were collected, and the levels of the various chemokines and cytokines were measured by multiplex enzyme-linked immunosorbent

assay. All the age-matched, non-TB progressors were healthy, nonsmoking and nonalcoholic and were without any immunosuppressive conditions at baseline and at follow-up. The data from the smoking and alcoholic progressors were not included. The p values were determined using one-way ANOVA with Tukey's multiple comparisons test. The mean values, SEM, and p values are shown.

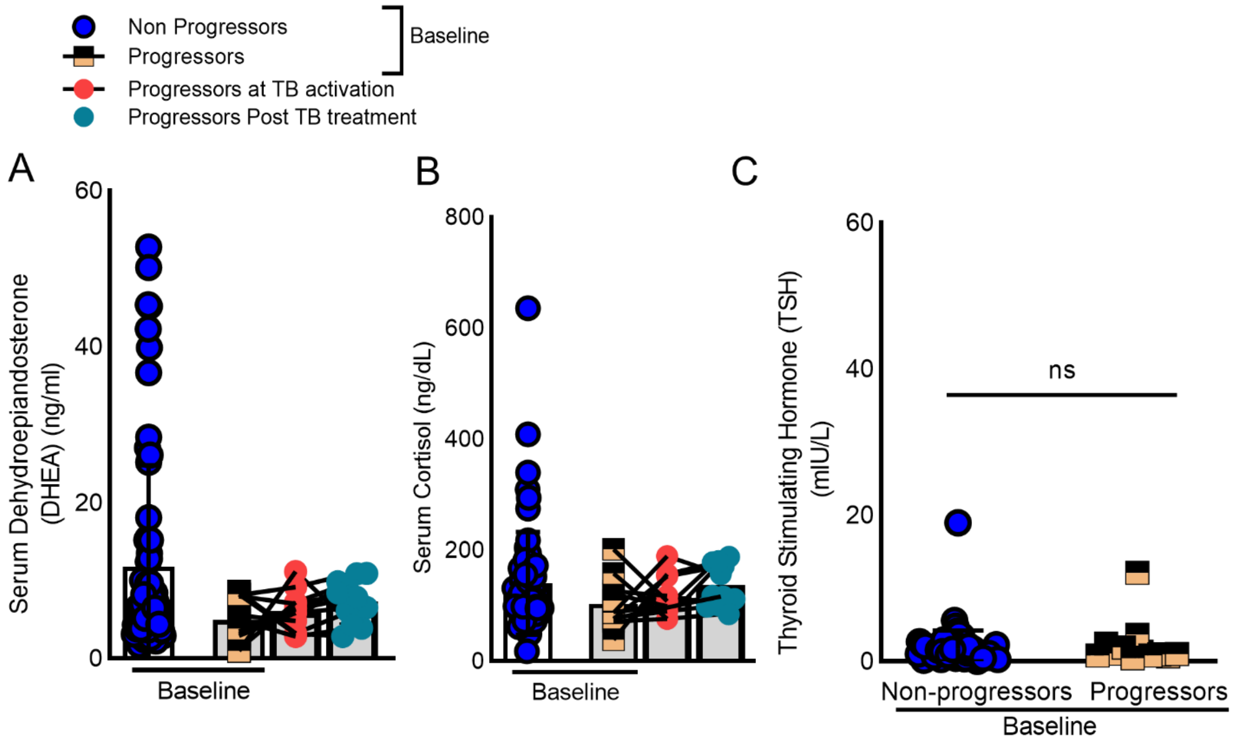

Figure S4. Thyroid stimulating hormone (TSH) levels in the serum of HHCs from TB patients. The serum (A) Dehydroepiandrosterone (DHEA) (B) cortisol and (C) thyroid stimulating hormone (TSH) levels of the non-TB progressors (n = 67) and TB progressors (n = 12) at baseline were quantified by ELISA. P values were derived by using an unpaired t test. The mean values, SD, and p values are shown.

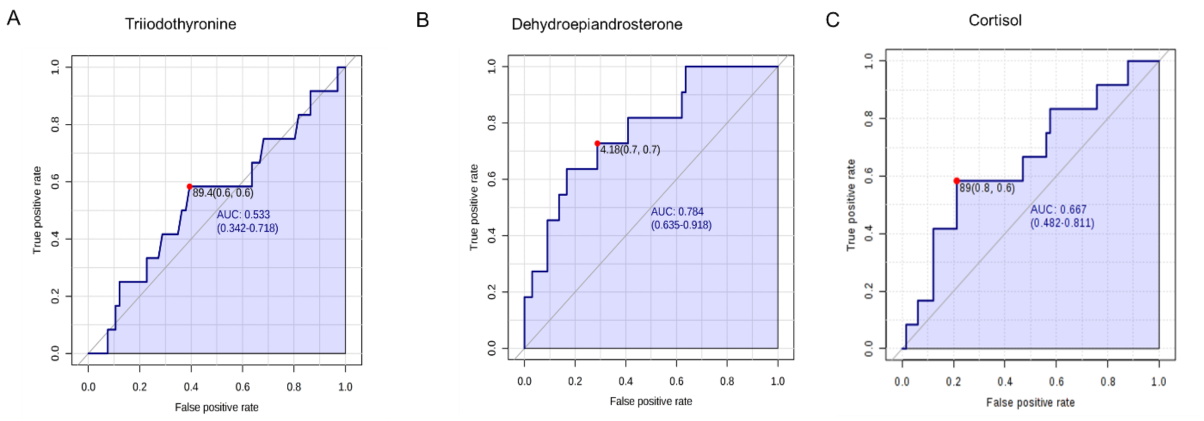

Figure S5. Receiver operating characteristic (ROC) curves for the hormone values obtained from nonprogressors and progressors at baseline. The plot shows the ROC curves for the hormones (A) T3, (B) DHEA and (C) cortisol measured in the serum of nonprogressors and progressors at baseline. Each ROC shows the points corresponding to the true positive rate (sensitivity) and false positive rate (specificity) of the model predictions.

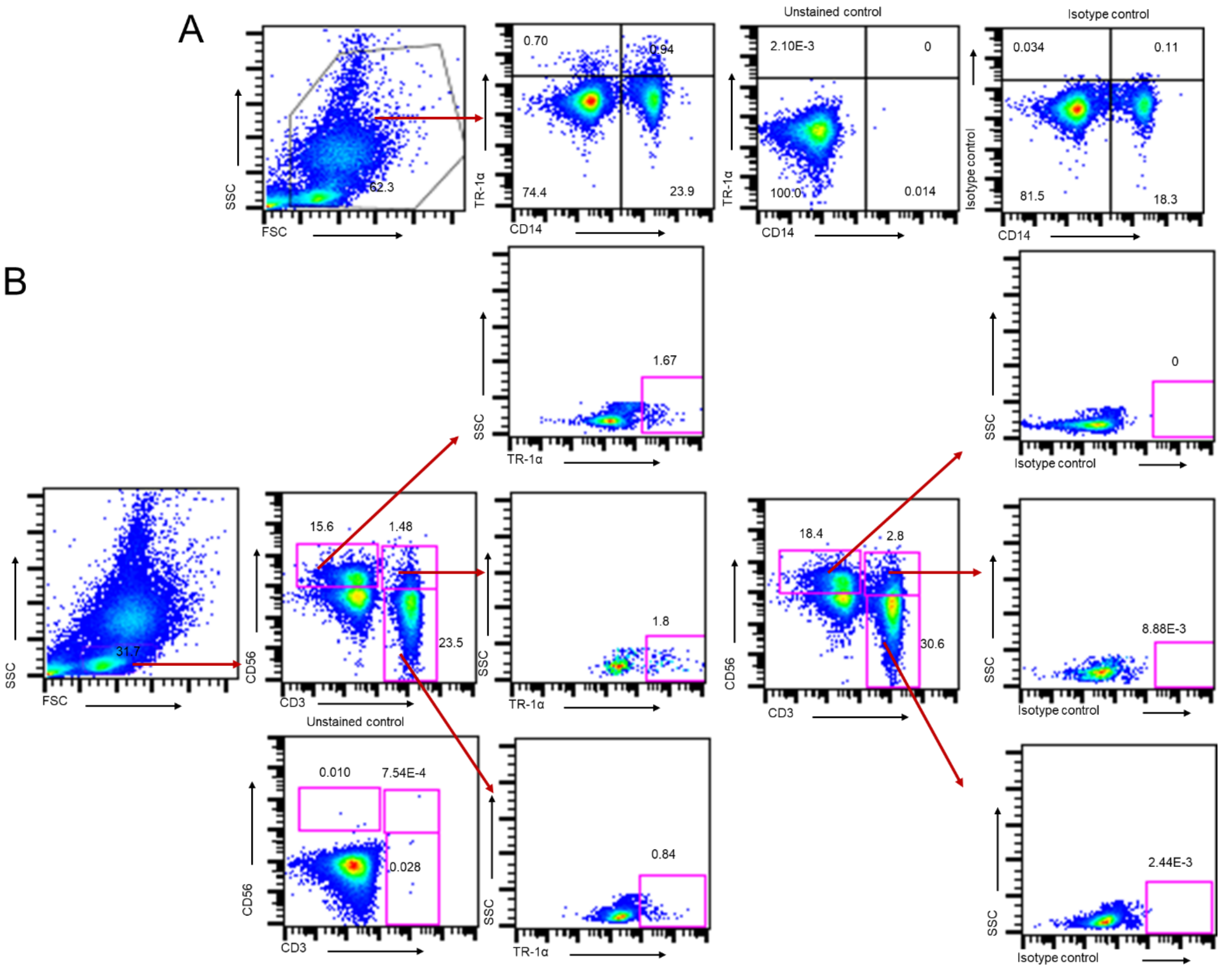

Figure S6. Thyroid receptor (TR) 1α expression in γ-Mtb-stimulated cells. Freshly isolated PBMCs from 6 healthy individuals (age range: 18-30 years) were cultured with or without γ-irradiated Mtb H37Rv (γ-Mtb, 10 μg/mL) for 120 hours. A representative image of the gating strategies used for TR 1α+ cells among (A) monocytes, (B) NK cells, T cells, and NKT cells is shown.

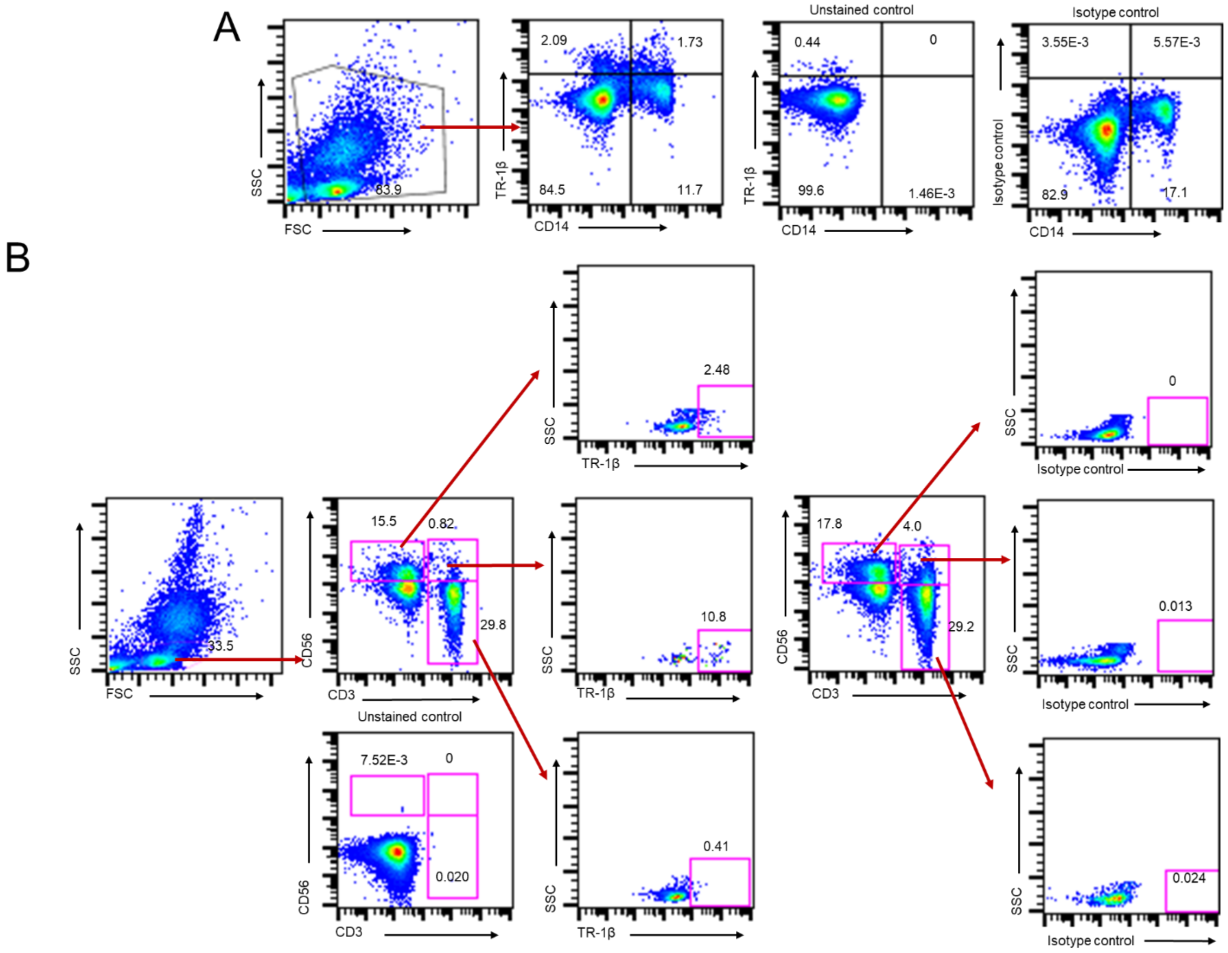

Figure S7. Thyroid receptor (TR) 1β expression in γ-irradiated Mtb-stimulated cells. Freshly isolated PBMCs from 6 healthy individuals (age range: 18-30 years) were cultured with or without γ-irradiated Mtb H37Rv (γ-Mtb, 10 μg/mL) for 120 hours. A representative image of the gating strategies for TR 1β+ cells among (A) monocytes, (B) NK cells, T cells, and NKT cells are shown.

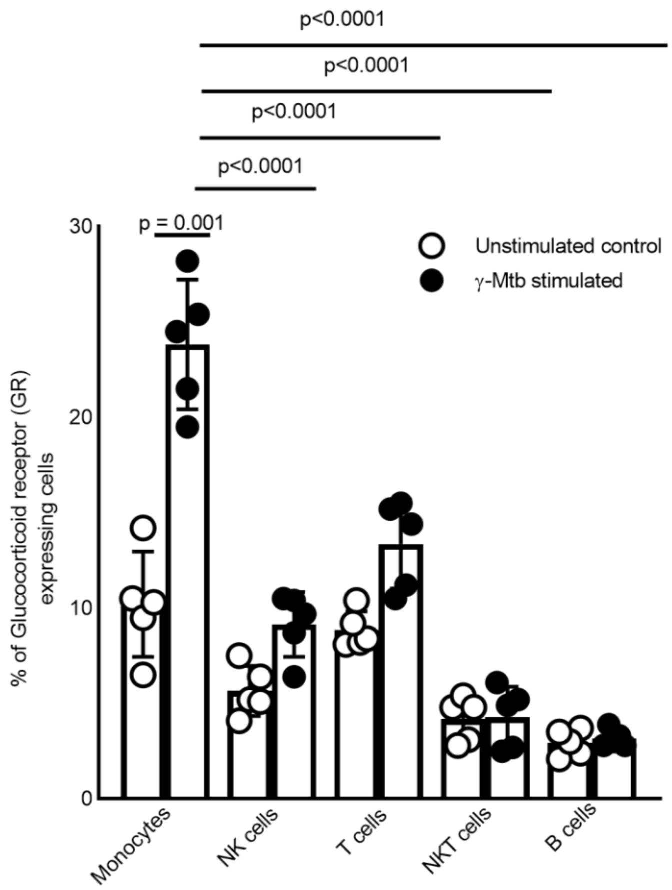

Figure S8. Glucocorticoid (GR1) receptor expression in γ-irradiated Mtb-stimulated cells. Freshly isolated PBMCs from 5 healthy individuals (age range: 18-30 years) were cultured with or without γ-irradiated Mtb H37Rv (γ-Mtb, 10 µg/mL) for 120 hours. The percentages of GR1+ cells among monocytes, NK cells, T cells, NKT cells and B cells were determined by flow cytometry. The p values were determined using one-way ANOVA with Bonferroni's multiple comparisons test. The mean values, SD and p values are shown.

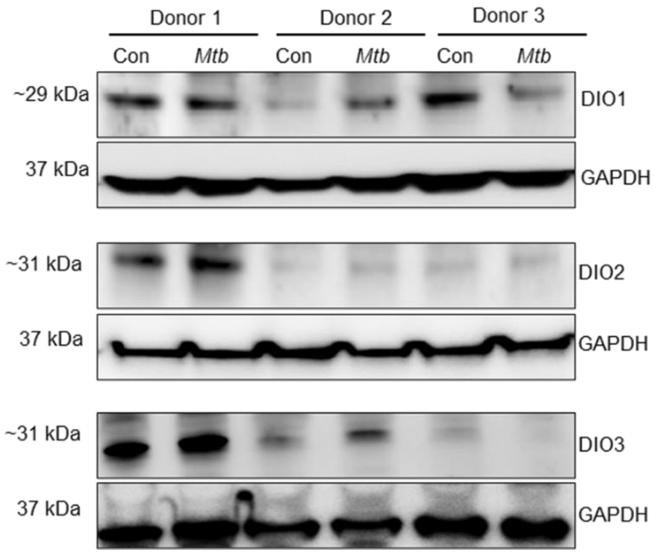

Figure S9. Deiodinases expression in human monocyte derived macrophages. MDM's of healthy volunteers (n = 3) were infected with *Mtb* H37Rv and protein lysates were collected after 72 h of infection using M-PER (mammalian protein extraction reagent) with protease and phosphatase inhibitors cocktail. The expression of DIO1, DIO2 and DIO3 were determined by western blotting.

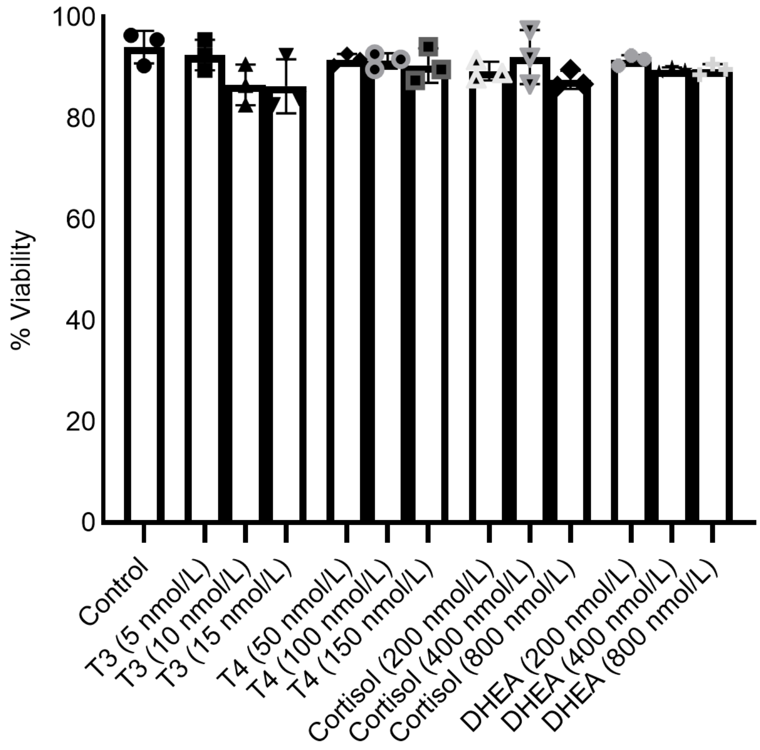

Figure S10. Cytotoxic effect of hormones on MDMs. MDMs were cultured in the presence of triiodothyronine (T3) (5, 10, 15 nmol/L), thyroxine (T4) (50, 100, 150 nmol/L), dehydroepiandrosterone (DHEA) (200, 400, 800 µg/dl) or cortisol (200, 400, 800 nmol/L) for 5 days. The percentage of MDM viability was estimated by using the MTT assay. The data are the mean ± SD for three separate experiments.

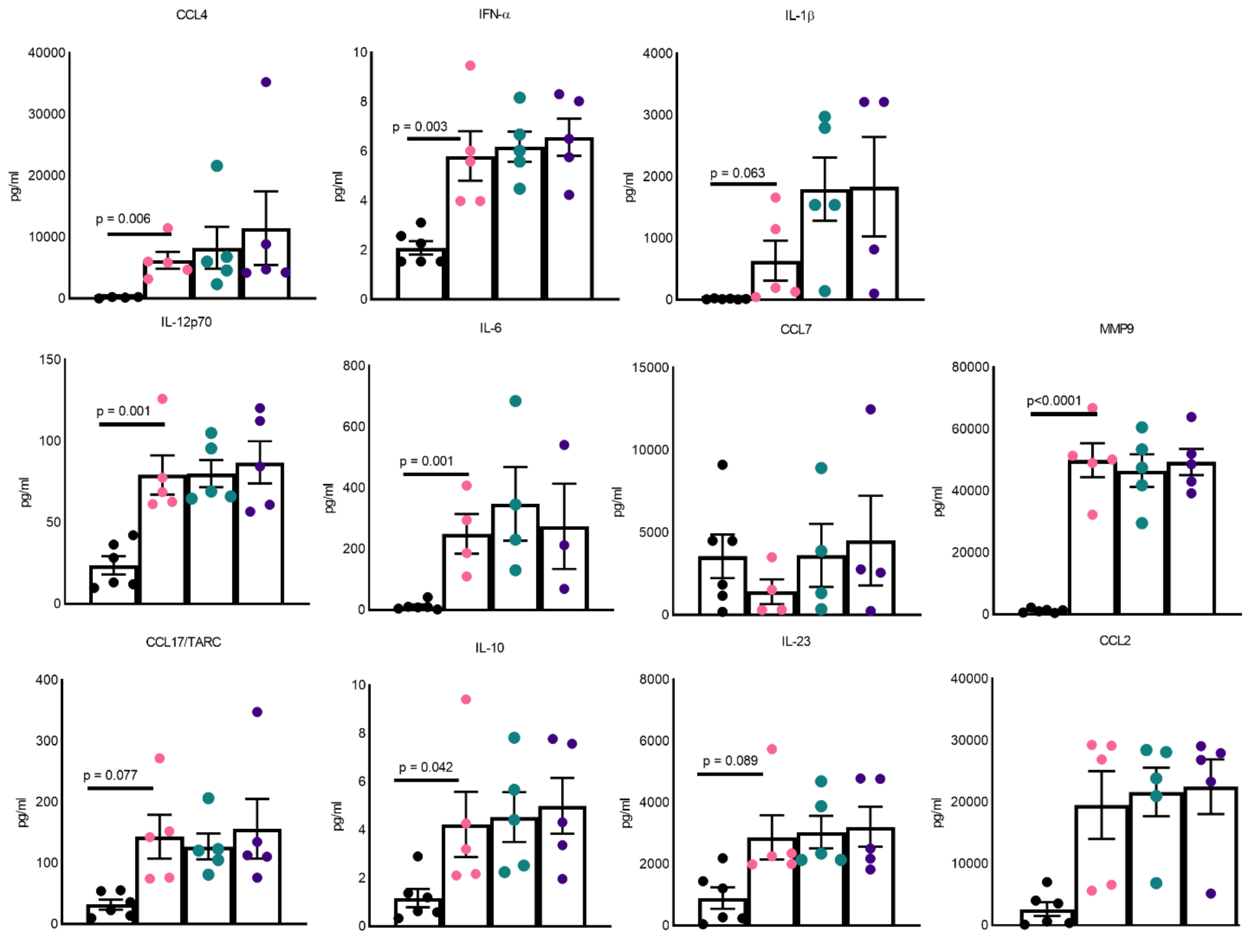

Figure S11. Thyroid hormones induce cytokine production by Mtb-infected MDMs. Freshly isolated MDMs from 18- to 30-year-old donors (n = 5) were infected with H37Rv at an MOI of 2.5, as described in the methods section. Some of the infected MDMs were cultured in the presence of T3 (15 nmol/L) or T4 (150 nmol/L) for 5 days. The supernatants were aspirated, and cytokine and chemokine production were measured by multiplex ELISA. The p values were determined using one-way ANOVA with Bonferroni's multiple comparisons test. The mean values, SD and p values are shown.

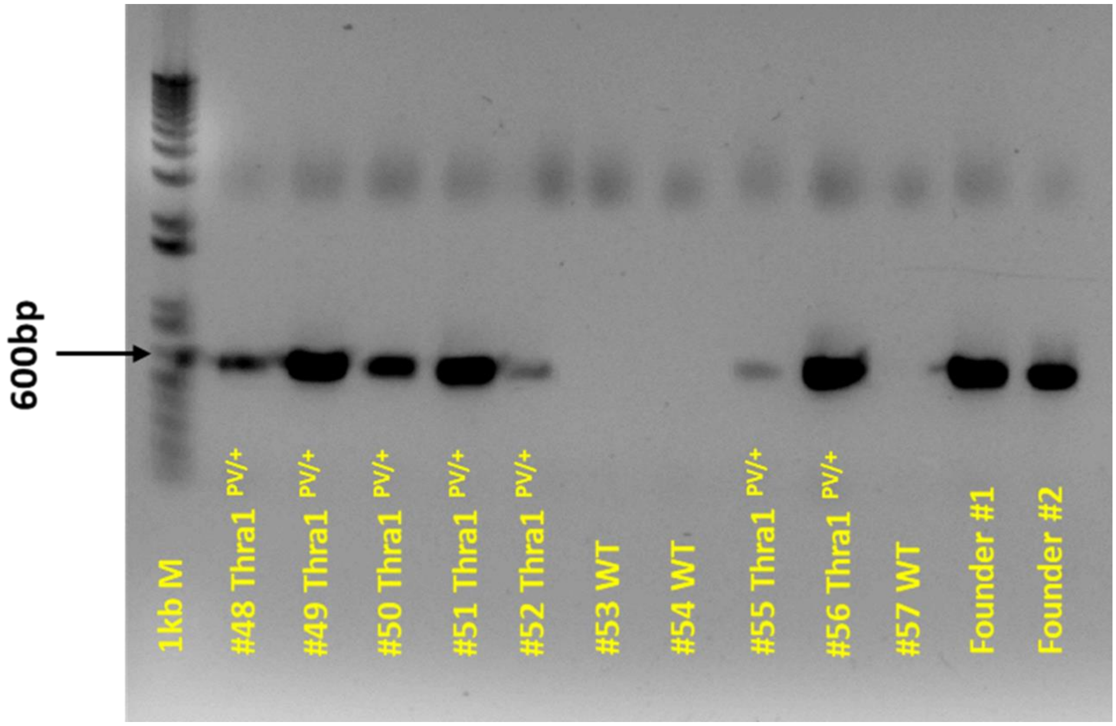

Figure S12. Genotyping of TRα1$^{PV/+}$ mice by PCR. Thyroid receptor TRα1-mutant mice were crossbred with C57BL/6 (wild type) mice to obtain homozygous TRα1$^{PV/+}$ mutant mice. Using the mouse alpha PV gPCR genotyping primer pairs, a 586-bp PCR product was confirmed in TRα1$^{PV/+}$ mice (#48-52, 55 and 56) with the respective founders (Founder #1 and Founder #2). However, the 586-bp PCR product was absent in wild-type littermates (#53, 54 and 57).

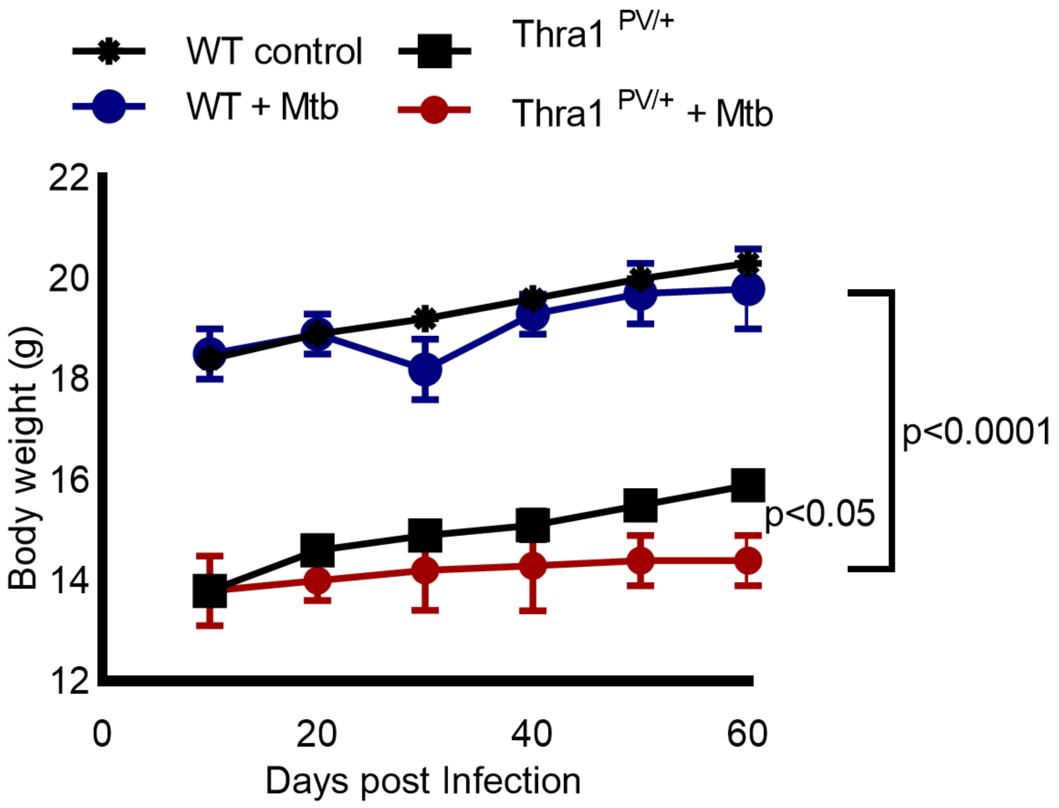

Figure S13. Body weight of TRα1$^{PV/+}$ mice. Wild-type (WT) and Thra1 $^{PV/+}$ mice (both C57BL/6 background) were infected with ~100 CFU of *Mtb* H37Rv by aerosol inhalation. At different time points after Mtb infection, the weight was determined. Five mice per group were used. The p values were determined using an independent t-test. Data are expressed as the mean ± SD.

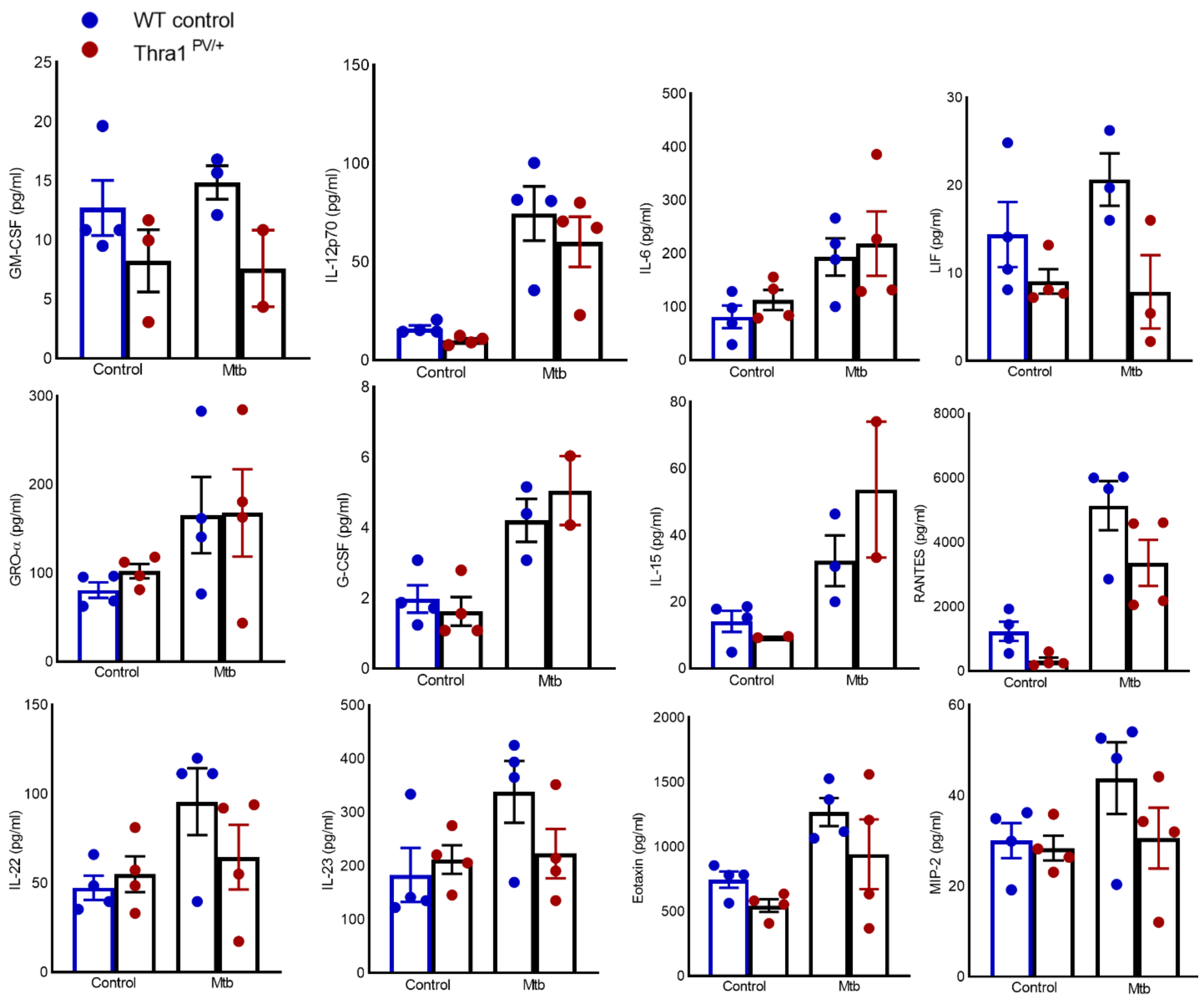

Figure S14. Cytokine and chemokine levels in Thra1 $^{PV/+}$ mice during Mtb infection. WT and Thra1 $^{PV/+}$ mice (both C57BL/6 background) were infected with ~100 CFU of *Mtb* H37Rv by aerosol inhalation. At 1 month post infection (p.i), lung homogenates from uninfected control and Thra1 $^{PV/+}$, Mtb-infected control, and Thra1 $^{PV/+}$ mice were collected, and the cytokine and chemokine levels were measured by multiplex ELISA. The p values were determined using one-way ANOVA with Bonferroni's multiple comparisons test. Four mice per group were used. Data are expressed as the mean ± SD.

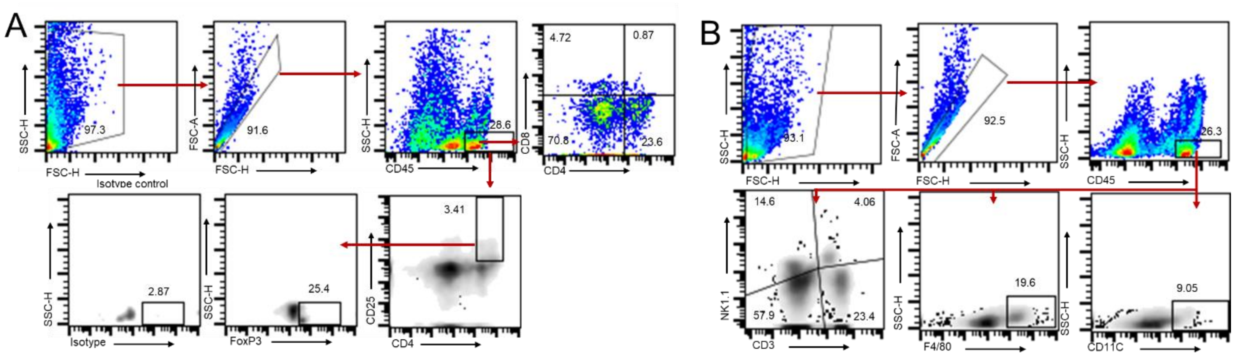

Figure S15. Gating strategy for the identification of various immune cell populations in mouse lungs. WT and Thra1 $^{PV/+}$ mice (both C57BL/6 background) were infected with ~100 CFU of Mtb H37Rv by aerosol inhalation. One-month post Mtb infection, a single cell suspension of lung tissue was prepared, and flow cytometry was performed. The flow cytometry gating strategies for various immune cell populations (A). CD45, CD4, CD8 and Tregs (B). NK cells, F4/80 and CD11c+ cells are shown.

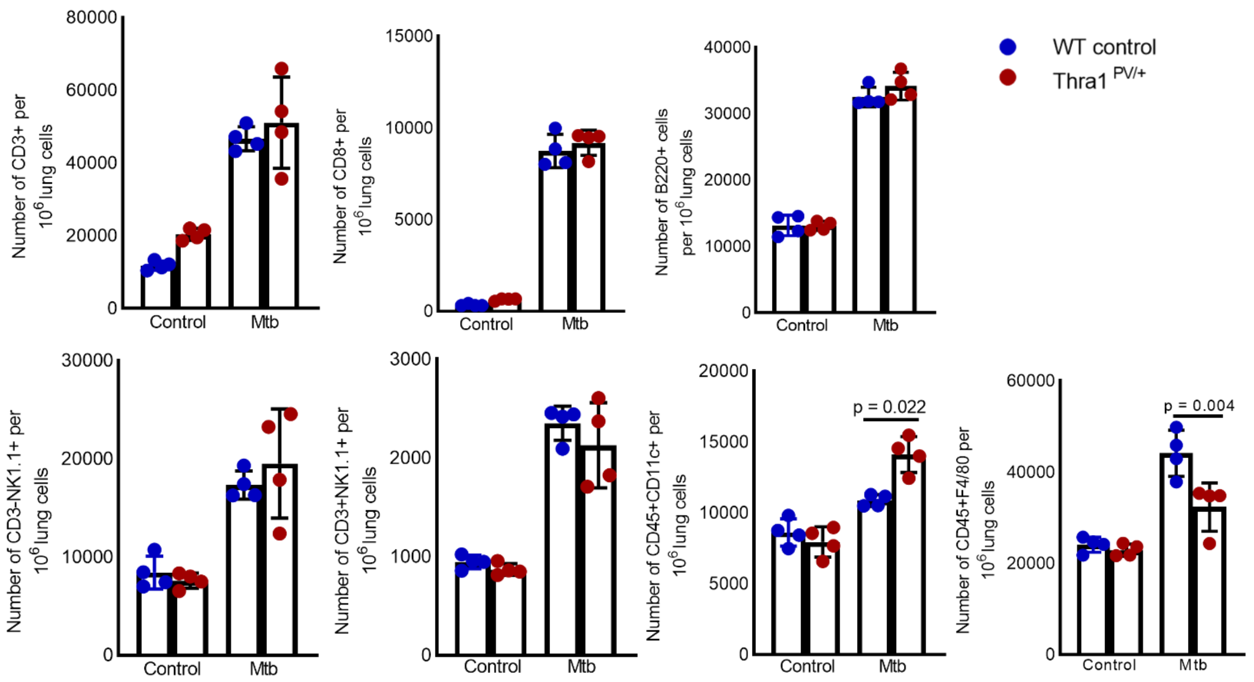

Figure S16. Populations in the lungs of Mtb-infected Thra1$^{PV/+}$ mice. WT and Thra1 $^{PV/+}$ mice (both C57BL/6 background) were infected with ~100 CFU of *Mtb* H37Rv by aerosol inhalation. At 1-month p.i., the numbers of various types of immune cells were measured by flow cytometry. Four mice per group were used. The p values were determined using one-way ANOVA with Bonferroni's multiple comparisons test. Data are expressed as the mean ± SD.

Table S1. HHC screening and enrollment.

Number of HHCs screened for the study	**1039**
Number enrolled into the study	**839**
Percentage of enrollment	**80.75**
**Recorded reasons for screening failure**	
Did not have significant exposure to the index case	**20**
History of TB disease	**21**
Signs and symptoms consistent with active TB	**15**
Plans to move out of the area during the study period	**29**
Does not consent to the collection of samples	**33**
Not interested in participating in the study	**19**
Unwilling to provide a reason	**31**
Other comorbidities	**22**
Pregnancy (women)	**10**

Table S2. HHCs lost to follow-up.

Number enrolled into the study	**839**
Number of HHCs lost to follow-up	**138**
Percentage of HHCs lost to follow-up	**16.4**
**Reasons for lost to follow-up**	
HHC no longer interested in participating in the study	**10**
Moved out of the area	**23**
Unwilling to provide a reason	**15**
Unwilling to participate due to death of the index case	**26**
Did not see any direct benefit from the study	**32**
Lost contact with the study staff	**31**
HIV-positive during follow-up	**1**

Table S3. Demographics and characteristics of the HHCs that progressed to active TB during the 2-year follow-up study.

S. No	Age	Gender	TB diagnosis	Baseline LTBI Status	Smoking	Alcohol	Any other comorbidities	History of BCG vaccination
1	27	Female	At 24th Month	Negative	Never	Never	-	Yes
2	28	Female	At 12th Month	Positive	Never	Never	-	Yes
3	18	Male	At 8th Month	Positive	Never	Never	-	Yes
4	18	Male	At 8th Month	Negative	Current	Current	-	Not known
5	43	Female	At 4th Month	Negative	Never	Never	Asthma	Yes
6	32	Male	At 12th Month	Positive	Never	Current	-	No
7	26	Male	At 4th Month	Positive	Never	Never	-	Yes
8	19	Male	At 16th Month	Positive	Current	Current	-	Yes
9	18	Female	At 24th Month	Negative	Never	Never	-	Yes
10	15	Male	At 16th Month	Negative	Never	Never	-	Yes
11	45	Female	At 12th Month	Positive	Never	Never	-	Yes
12	27	Female	At 16th Month	Negative	Never	Never	-	Yes
13	18	Male	At 12th Month	Negative	Never	Never	-	Not known
14	16	Female	At 4th Month	Negative	Never	Never	-	Yes
15	15	Male	At 4th Month	Positive	Never	Never	-	Not known
16	17	Female	At 4th Month	Positive	Never	Never	-	Yes
17	15	Male	At 4th Month	Positive	Never	Never	-	Yes

Table S4. Primer sequences.

**Wild type Thra**	**F 5-TCTTGTCCTCGGGCCTCATGCC-3**
	**R 5-CTCTGGCCGCCTGAGGCTTTAG-3**
**Mutant Thra**	**F 5-CTGTGCGTGGACAAGATCGAGA-3**
	**R 5-CTGACCGCTTCCTCGTGCTTTACG-3**

Table S5. Resource

REAGENT or RESOURCE	SOURCE	IDENTIFIER
**Antibodies**		
Cytokine & Chemokine 34-Plex Human ProcartaPlex™ Panel 1A	Thermo Fisher Scientific	EPX340-12167-901
Human magnetic Luminex Assays	RD Systems	LXSAHM-20
Anti human CD 16 PE antibody	BD Pharmingen	Cat#555407 Clone 3G8
Anti human CD 56 APC antibody	BD Pharmingen	Cat#555518<br>Clone B159
Anti human CD 27 PE antibody	BD Pharmingen	Cat#555441<br>Clone M-T271
Anti human CD3 PerCP antibody	BD Bioscience	Cat#347344<br>Clone SK7
Anti human CD3 FITC antibody	BD Pharmingen	Cat#555332<br>Clone UCHT1
Anti human IL-21 R PE antibody	BD Pharmingen	Cat#560264<br>Clone 17A12
Anti human CD4 FITC antibody	BD Pharmingen	Cat#555346<br>Clone RPA-T4
Anti human CD25 APC antibody	BD Pharmingen	Cat#555434<br>Clone M-A251
Anti human Foxp3 PE antibody	BD Pharmingen	Cat#560046<br>Clone 259D/C7
Anti human PD1 FITC antibody	BIOLEGEND	Cat#329904<br>EH12.2H7
Anti human CD 14 Per CP antibody	BIOLEGEND	Cat#325632<br>HCD14

Anti human CCR7 FITC antibody	BIOLEGEND	Cat#353216<br>G043H7
Anti human CXCR6 Cy7 antibody	BIOLEGEND	Cat#356012<br>K041E5
Anti-Thryroid hormone receptor beta antibody Alexa flour 488 conjugated	Bioss	Bs-11440R-A488
Anti-Thryroid hormone receptor antibody Alexa flour 488 conjugated	Bioss	Bs-6221R-FITC
PE anti-human GPR83 Antibody	Biolegend	Cat# 363306<br>K07JP05
IL-1 alpha Monoclonal Antibody (ALF-161), Functional Grade,	eBioscience	Cat# 16-7011-85
Anti-IL-1 beta antibody	abcam	Cat# ab2105
Anti-Thyroid Hormone Receptor antibody	abcam	Cat# ab53729
**Biological Samples**		
Human PBMCs	Primary/Healthy volunteers	
Human Plasma	Primary/Healthy volunteers	
Human Serum	Primary/Healthy volunteers	
**Chemicals, Peptides, and Recombinant Proteins**		
RPMI (1640)	Life Technologies	
BSA	Sigma	A7030

Triiodo-L-Thyronine (T3)	Sigma	T2877
L-Thyroxine	Sigma	T2376
Cortisol	Sigma	C-113
Dehydroepiandrosterone (DHEA)	Sigma	D-063
Macrophage-SFM (1X)	Thermo Fisher Scientific	12065074
penicillin/streptomycin	Sigma	P4333
BD BBL™ Middlebrook OADC Enrichment	BD	B11886
Sodium dodecyl sulfate	Sigma	436143
Mycobacterium *tuberculosis* ESAT-6 peptides	BEI Resources, USA	NR-34824
Mycobacterium *tuberculosis* CFP10 peptides	BEI Resources, USA	NR-34825
Mycobacterium tuberculosis, Strain H37RV, Gamma-Irradiated Whole Cells	BEI Resources, USA	NR-49098
**Software and Algorithms**		
ImageJ (v1.5) (Schneider et al., 2012) https://imagej.nih.gov/ij/		
Prism 8.0 Graphpad Software N/A		
FlowJo (v10.1) Treestar https://www.flowjo.com/		
Graphical abstract created with BioRender.com	License No: E93E8064-0005	Biorender https://biorender.com/
Other		
TSH measurement	TSH ELISA Merilisa i	Meril Diagnostics<br>(REF TSIELI-01)
T4 measurement	T4 ELISA Merilisa i	Meril Diagnostics (REF: ITFELI-01)

Histopaque-1077	Sigma-Aldrich	Cat#10771-500 mL
